# Effect of Values Affirmation on Reducing Racial Differences in Adherence to Hypertension Medication: The HYVALUE Randomized Clinical Trial

**DOI:** 10.1001/jamanetworkopen.2021.39533

**Published:** 2021-12-16

**Authors:** Stacie L. Daugherty, Laura Helmkamp, Suma Vupputuri, Rebecca Hanratty, John F. Steiner, Irene V. Blair, L. Miriam Dickinson, Julie A. Maertens, Edward P. Havranek

**Affiliations:** 1 Division of Cardiology, Department of Medicine, University of Colorado School of Medicine, Aurora; 2 Adult and Children Center for Outcomes Research and Delivery Sciences, University of Colorado, Aurora; 3 Colorado Cardiovascular Outcomes Research Group, University of Colorado, Aurora, Denver; 4 Mid-Atlantic Permanente Research Institute, Kaiser Permanente Mid-Atlantic States, Rockville, Maryland; 5 Department of Medicine, Denver Health and Hospital Authority, Denver, Colorado; 6 Institute for Health Research, Kaiser Permanente Colorado, Denver; 7 Department of Psychology and Neuroscience, University of Colorado Boulder, Boulder; 8 Department of Family Medicine, University of Colorado School of Medicine, Aurora

## Abstract

**Importance:**

Stereotype threat, or the fear of confirming a negative stereotype about one’s social group, may contribute to racial differences in adherence to medications by decreasing patient activation to manage chronic conditions.

**Objective:**

To examine whether a values affirmation writing exercise improves medication adherence and whether the effect differs by patient race.

**Design, Setting, and Participants:**

The Hypertension and Values trial, a patient-level, blinded randomized clinical trial, compared an intervention and a control writing exercise delivered immediately prior to a clinic appointment. Of 20 777 eligible, self-identified non-Hispanic Black and White patients with uncontrolled hypertension who were taking blood pressure (BP) medications, 3891 were approached and 960 enrolled. Block randomization by self-identified race ensured balanced randomization. Patients enrolled between February 1, 2017, and December 31, 2019, at 11 US safety-net and community primary care clinics, with outcomes assessed at 3 and 6 months. Analysis was performed on an intention-to-treat basis.

**Interventions:**

From a list of 11 values, intervention patients wrote about their most important values and control patients wrote about their least important values.

**Main Outcomes and Measures:**

The primary outcome of adherence to BP medications was measured using pharmacy fill data (proportion of days covered >90%) at baseline, 3 months, and 6 months. The secondary outcome was systolic and diastolic BP. Patient activation to manage their health was also measured.

**Results:**

Of 960 patients, 474 (286 women [60.3%]; 256 Black patients [54.0%]; mean [SD] age, 63.4 [11.9] years) were randomly assigned to the intervention group and 486 (288 women [59.3%]; 272 Black patients [56.0%]; mean [SD] age, 62.8 [12.0] years) to the control group. Baseline medication adherence was lower (318 of 482 [66.0%] vs 331 of 412 [80.3%]) and mean (SE) BP higher among Black patients compared with White patients (systolic BP, 140.6 [18.5] vs 137.3 [17.8] mm Hg; diastolic BP, 83.9 [12.6] vs 79.7 [11.3] mm Hg). Compared with baseline, pharmacy fill adherence did not differ between intervention and control groups at 3 months (odds ratio [OR], 0.91 [95% CI, 0.57-1.43]) or at 6 months (OR, 0.86 [95% CI, 0.53-1.38]). There were also no treatment effect differences in pharmacy fill adherence by patient race (Black patients at 3 months: OR, 1.08 [95% CI, 0.61-1.92]; at 6 months: OR, 1.04 [95% CI, 0.58-1.87]; White patients at 3 months: OR, 0.68 [95% CI, 0.33-1.44]; at 6 months: OR, 0.55 [95% CI, 0.24-1.27]). Immediately after the intervention, the median patient activation was higher in intervention patients than in control patients, but this difference was not statistically significant in an unadjusted comparison (75.0 [IQR, 65.5-84.8] vs 72.5 [IQR, 63.1-80.9]; *P* = .06). In adjusted models, the Patient Activation Measure score immediately after the intervention was significantly higher in the intervention patients than in control patients (mean difference, 2.3 [95% CI, 0.1-4.5]).

**Conclusions and Relevance:**

A values affirmation intervention was associated with higher patient activation overall but did not improve adherence or blood pressure among Black and White patients with hypertension.

**Trial Registration:**

ClinicalTrials.gov Identifier: NCT03028597

## Key Points

**Question:**

Does a writing intervention focused on affirming one’s personal values improve adherence to hypertension medication?

**Findings:**

In this randomized clinical trial including 960 adults with hypertension, patients who received a values affirmation intervention prior to a primary care visit had no significant change in 3-month or 6-month medication adherence or blood pressure compared with controls. Intervention response was comparable in self-identified Black and White patients.

**Meaning:**

A brief writing exercise that affirms individual values did not significantly improve adherence to medication or blood pressure for Black or White patients with hypertension.

## Teaser

This randomized clinical trial examines whether a values affirmation writing exercise improves medication adherence and whether the effect differs by patient race and ethnicity.

## Introduction

In the US, Black individuals have a higher prevalence of uncontrolled hypertension than White individuals.^1^ Rates of adherence to hypertension medication are lower in Black individuals than in White individuals with hypertension in the US, and nonadherence contributes to racial and ethnic differences in hypertension control.^2,3,4,5^ Interventions to improve medication adherence might reduce racial and ethnic disparities in hypertension outcomes.

Stereotype threat, or the threat of being identified with a negative stereotype about one’s social group (eg, race and ethnicity) may contribute to racial and ethnic differences in medication adherence.^6,7^ For example, if a Black patient is fearful of being subjected to the negative stereotype of being unintelligent, they may not ask questions or engage during their visit. They may leave the clinic without information and not feel activated to manage their blood pressure (BP).^6,8^ Patients can also experience general psychological threats to their sense of self unrelated to race and ethnicity. Those with uncontrolled hypertension might feel subject to a stereotype of being nonadherent.^8,9^ Interventions to support patients who are subject to stereotype threat might improve patient adherence and improve health outcomes.^6,9^

Values affirmation interventions target stereotype threat and have reduced racial disparities in education^10,11^ and improved patient-clinician communication.^12^ These interventions ask patients to reflect on and write about values that are most important to them, such as family or religion.^13^ This process may remind patients of sources of support and reassurance outside the medical context and allow them to be more open to health information despite potential stereotype threat.^14,15,16^ The Hypertension and Values (HYVALUE) randomized clinical trial tested the hypothesis that values affirmation disrupts the negative effects of stereotype threat on the clinical interaction, improves patient activation, and improves medication adherence.^6^ Intervention effects were compared in self-identified non-Hispanic Black and White patients to test whether the threat ameliorated was that of racial stereotyping.

## Methods

### Design, Setting, and Participants

The HYVALUE rationale and protocol were published^6^ (trial protocol in Supplement 1). In brief, the trial was a multicenter patient-level, randomized, controlled, blinded (study and medical team blinded) clinical trial. Patients enrolled between February 1, 2017, and December 31, 2019, at 11 primary care clinics from 3 sites: Denver Health, a safety-net system in Denver, Colorado; Kaiser Permanente Colorado, a community system in Denver; and Kaiser Permanente Mid-Atlantic States, a community system in Maryland. Institutional review boards at all 3 sites approved study procedures, and written consent was obtained from all patients. The study followed the Consolidated Standards of Reporting Trials (CONSORT) reporting guideline for randomized clinical trials.^17^

Patients were eligible if they had a primary or secondary *International Statistical Classification of Diseases and Related Health Problems, Tenth Revision* code diagnosis of hypertension in the preceding 24 months and uncontrolled BP, which was defined as a systolic BP of 140 mm Hg or more or diastolic BP of 90 mm Hg or more at least once during the preceding 12 months.^18,19^ Additional inclusion criteria were age 21 years or older, self-identified race and ethnicity of non-Hispanic Black or non-Hispanic White, receiving antihypertensive medication(s) from the health system’s pharmacy, and the ability to read and write English. Participants self-reported gender. Patients who were pregnant, had pregnancy-related hypertension, or had end-stage renal disease were excluded.

### Randomization and Intervention

Patients were randomized into intervention and control groups by race and health system, using block randomization. The randomization was created by the data coordinating center analyst (L.H.) prior to patient recruitment using SAS, version 9.4 (SAS Institute Inc). Study personnel and the patient’s clinical team were blinded to treatment group.^6^

All patients were randomized to either a values affirmation intervention or a control writing exercise, completed immediately before a scheduled primary care appointment. Both writing exercises asked patients to reflect on 11 values (Box). Intervention group patients picked 2 or 3 values that were most important to them and wrote a few sentences to describe why these values were important to them. Control group patients picked 2 or 3 values that were least important to them and wrote a few sentences about why these values might be important to someone else.

Box. **List of Values for the Intervention and Control Exercises**

1. Sense of humor
2. Religious values
3. Relationships with friends or family
4. Music
5. Politics
6. Membership in a community or social group
7. Living in the moment
8. Independence
9. Creativity
10. Artistic ability
11. Athletic ability

### Outcomes

The primary outcome was antihypertensive medication adherence, measured using pharmacy fills at baseline, 3 months, and 6 months. Pharmacy fill adherence measured the medication supply obtained during the period of observation using the proportion of days covered (PDC).^20^ Second, the 3-item self-report survey measured nonadherence during the previous 7 days (agreement with any question [eg, “I missed or skipped at least 1 dose”] indicated nonadherence).^21^ Third, pill count adherence measured the number of pills missing, divided by the number of expected pills taken since the last refill (range, 0.0-1.0).^22^ For each patient, pill count and PDC adherence were calculated for each antihypertensive drug and averaged across drugs into a summary measure.^20^

The secondary outcome was study-measured BP at baseline, 3 months, and 6 months. Study BPs were measured by study staff per study protocol and guideline-recommended procedures.^6,19^

### Patient Activation

Patient activation was measured using the 13-item Patient Activation Measure (PAM).^23^ Each patient completed the PAM immediately after the baseline clinic visit (after intervention delivery) and repeated the PAM at each visit. PAM scores range from 0 to 100, with higher scores indicating greater activation for engagement in health care. The PAM assesses knowledge, skills, beliefs, and behaviors that a patient needs to manage chronic illness.^23^

### Fidelity of Intervention

We evaluated the writing exercises after study completion to assess patient fidelity to instruction and self-affirmation.^24^ We evaluated the fit of the intervention by asking about the salience of the 11 values listed (eMethods 1 in Supplement 2).

### Statistical Analysis

Using intention-to-treat principles, the primary analysis was at the patient level using generalized linear mixed-effects models that assessed adherence over time by study group and patient race. The 3 adherence outcomes were analyzed separately. Fixed effects included time, race, study group, and study site with random effects of clinician and patient (eMethods 2 in Supplement 2). Pharmacy fill adherence was skewed (baseline median PDC, 0.98 [IQR, 0.88-1.00]) and dichotomized as more than 90% PDC (adherent) or 90% or less PDC (nonadherent).^25^ Self-reported adherence was dichotomized into nonadherent (score ≥2 on any item) and adherent.^21^ Pill count adherence and BP were normally distributed and treated as continuous variables. Assumptions of all models were verified.

Prior to analyses, we examined the data to determine patterns of missingness and found no evidence of nonignorable missingness.^26,27,28^ For pharmacy fills, 6.9% (66 of 960), 10.1% (97 of 960), and 12.9% (124 of 960) had missing data at baseline, 3 months, and 6 months, respectively. Owing to incomplete surveys or missed study visits, 1.9% (18 of 960), 37.0% (355 of 960), and 34.3% (329 of 960) had missing self-reported adherence at baseline, 3 months, and 6 months, respectively. Patients often forgot to bring their medications to study visits; 18.5% (178 of 960), 51.9% (498 of 960), and 56.3% (540 of 960) had missing pill counts at baseline, 3 months, and 6 months, respectively. Trial-measured BP was missing for 1.0% (10 of 960) at baseline, 37.4% (359 of 960) at 3 months, and 44.9% (431 of 960) at 6 months. Missingness did not differ by study group. We used full conditional likelihood–based methods (generalized linear mixed models) that used all available data and adjusted for covariates that were associated with missingness in the final models.^26,27,28^ This approach has been shown to provide relatively unbiased results and “missing at random” (MAR).

The target enrollment was 1130 patients and assumed a final sample of 960 patients, giving more than 80% power to detect a between-group effect size of 0.26 or 4.7% difference in adherence between any 2 cells defined by race and study group.^6^ Recruitment was slower than anticipated, and 960 patients enrolled. All analysis used SAS, version 9.4. All *P* values were from 2-sided tests, and results were deemed statistically significant at *P* < .05.

A data and safety monitoring board met before study initiation, approved the protocol, and met at least yearly. The board monitored study conduct, data quality, and safety; no interim efficacy analyses were planned or conducted. On review of the results, the board released the data for publication.

## Results

### Patient Characteristics

Of the 20 777 patients who met eligibility criteria, study staff approached 3891. Of these, 2240 were not reached (nonworking telephone number, never answered, did not return message). Of the 1651 reached, 613 declined to participate and 78 did not complete an appointment. A total of 960 patients were randomized to the intervention (Figure). Compared with the eligible population, those who enrolled were more likely to be of White race, be from lower income census tracts, and have more comorbidities (eTable in Supplement 2).

**Figure.**
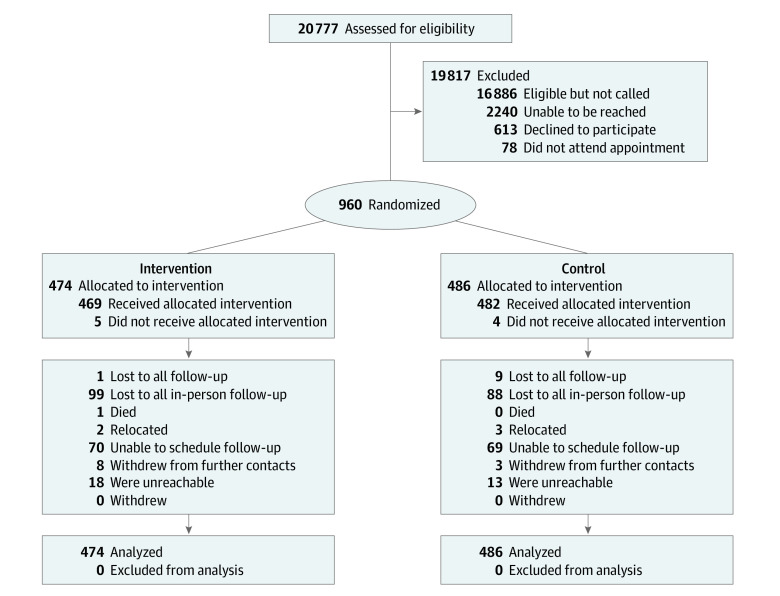
The Hypertension and Values (HYVALUE) Trial CONSORT Diagram Assessed for eligibility: Those who met all inclusion criteria over the study period based on electronic health record data. Eligible but not called: Lists of eligible patients exceeded study team ability to screen and call all patients. Received allocated intervention: Patient consented, completed written exercise, and saw primary care provider after intervention delivery. Did not receive allocated intervention: A total of 9 patients across the 2 study groups were enrolled on the day of a scheduled primary care visit, and the visit was canceled by the clinic after the patient was randomized. The visit was not able to be rescheduled within 1 week of randomization. Lost to all follow-up: Patient moved or otherwise relocated out of the health system and had no electronic follow-up data after study enrollment. Lost to all in-person follow-up: Patient had no in-person or telephone follow-up data. Patient relocated, did not want to schedule follow-up visit at that time, did not return messages, withdrew from further contact, or was unreachable for follow-up scheduling. Patient was unreachable for follow-up scheduling.

Of the 960 patients, 474 (286 women [60.3%]; 256 Black patients [54.0%]; mean [SD] age, 63.4 [11.9] years) were randomized to the intervention group and 486 (288 women [59.3%]; 272 Black patients [56.0%]; mean [SD] age, 62.8 [12.0] years) to the control group. Patient sociodemographic and clinical factors were balanced across the study groups (Table 1).

**Table 1.** Study Population Characteristics by Treatment Group and Patient Race

Characteristic^a^	White patients, No./total No. (%)		Black patients, No./total No. (%)	
	Control group (n = 214)	Treatment group (n = 218)	Control group (n = 272)	Treatment group (n = 256)
Gender, self-reported				
Male	100/214 (46.7)	96/217 (44.2)	93/270 (34.4)	89/256 (34.8)
Female	113/214 (52.8)	119/217 (54.8)	175/270 (64.8)	167/256 (65.2)
Transgender	0/214 (0.0)	1/217 (0.5)	2/270 (0.7)	0/256 (0.0)
Other gender	1/214 (0.5)	1/217 (0.5)	0/270 (0.0)	0/256 (0.0)
Age, y				
<45	11/214 (5.1)	10/218 (4.6)	25/272 (9.2)	18/256 (7.0)
45-54	21/214 (9.8)	24/218 (11.0)	45/272 (16.5)	52/256 (20.3)
55-64	55/214 (25.7)	47/218 (21.6)	113/272 (41.5)	84/256 (32.8)
65-74	70/214 (32.7)	87/218 (39.9)	64/272 (23.5)	73/256 (28.5)
≥75	57/214 (26.6)	50/218 (22.9)	25/272 (9.2)	29/256 (11.3)
Highest level of education				
<High school	7/209 (3.3)	8/216 (3.7)	20/270 (7.4)	14/255 (5.5)
High school diploma or equivalent	36/209 (17.2)	32/216 (14.8)	74/270 (27.4)	83/255 (32.5)
Some college, no degree	46/209 (22.0)	47/216 (21.8)	76/270 (28.1)	81/255 (31.8)
College degree	120/209 (57.4)	129/216 (59.7)	100/270 (37.0)	77/255 (30.2)
Employment status				
Employed	68/212 (32.1)	72/218 (33.0)	107/267 (40.1)	107/255 (42.0)
Unemployed	8/212 (3.8)	10/218 (4.6)	21/267 (7.9)	15/255 (5.9)
Retired	110/212 (51.9)	113/218 (51.8)	87/267 (32.6)	93/255 (36.5)
Disabled	26/212 (12.3)	23/218 (10.6)	52/267 (19.5)	40/255 (15.7)
Insurance status				
Medicaid	32/213 (15.0)	23/218 (10.6)	69/264 (26.1)	54/255 (21.2)
Medicare	90/213 (42.3)	100/218 (45.9)	55/264 (20.8)	70/255 (27.5)
Commercial	77/213 (36.2)	75/218 (34.4)	94/264 (35.6)	90/255 (35.3)
Uninsured or self-pay	1/213 (0.5)	2/218 (0.9)	1/264 (0.4)	0/255 (0.0)
Other	7/213 (3.3)	6/218 (2.8)	18/264 (6.8)	15/255 (5.9)
Medicaid and Medicare	6/213 (2.8)	12/218 (5.5)	27/264 (10.2)	26/255 (10.2)
Marital status				
Unmarried or unknown	98/212 (46.2)	99/217 (45.6)	96/261 (36.8)	90/251 (35.9)
Married	114/212 (53.8)	118/217 (54.4)	165/261 (63.2)	161/251 (64.1)
Living situation				
Lives alone	78/211 (37.0)	74/217 (34.1)	91/265 (34.3)	80/253 (31.6)
Lives with other(s)	133/211 (63.0)	143/217 (65.9)	174/265 (65.7)	173/253 (68.4)
Difficulty in paying for basics such as food, housing, medical care, and heating				
Very difficult	17/207 (8.2)	19/215 (8.8)	40/254 (15.7)	38/238 (16.0)
Somewhat difficult	49/207 (23.7)	52/215 (24.2)	92/254 (36.2)	71/238 (29.8)
Not difficult at all	141/207 (68.1)	144/215 (67.0)	122/254 (48.0)	129/238 (54.2)
No. of blood pressure medications				
1	106/211 (50.2)	121/215 (56.3)	150/269 (55.8)	123/255 (48.2)
2	72/211 (34.1)	60/215 (27.9)	66/269 (24.5)	83/255 (32.5)
3	24/211 (11.4)	22/215 (10.2)	33/269 (12.3)	34/255 (13.3)
≥4	9/211 (4.3)	12/215 (5.6)	20/269 (7.4)	15/255 (5.9)
Comorbidities^b^				
Ischemic heart disease	44/212 (20.8)	44/218 (20.2)	35/271 (12.9)	23/256 (9.0)
Cerebrovascular disease	20/212 (9.4)	24/218 (11.0)	23/271 (8.5)	22/256 (8.6)
Cardiac arrhythmia	51/212 (24.1)	54/218 (24.8)	37/271 (13.7)	31/256 (12.1)
Heart failure	34/212 (16.0)	28/218 (12.8)	25/271 (9.2)	25/256 (9.8)
Peripheral vascular disease	56/212 (26.4)	49/218 (22.5)	44/271 (16.2)	46/256 (18.0)
Type 1 or 2 diabetes	62/212 (29.2)	64/218 (29.4)	110/271 (40.6)	108/256 (42.2)
Renal failure	46/212 (21.7)	54/218 (24.8)	53/271 (19.6)	45/256 (17.6)
Depression	65/212 (30.7)	72/218 (33.0)	42/271 (15.5)	47/256 (18.4)
Current smoker	22/212 (10.4)	21/217 (9.7)	45/268 (16.8)	43/251 (17.1)

^a^ Less than 2% of patients were missing data on gender, educational level, employment, insurance, marital status, living situation, number of blood pressure medications, comorbidities, and blood pressure at baseline. “Difficulty in paying for basics” was missing for 5% of patients.

^b^ Based on *International Classification of Diseases, Ninth Revision* or *International Statistical Classification of Diseases and Related Health Problems, Tenth Revision* codes.

### Adherence Outcomes

#### Pharmacy Fill Adherence

At baseline, the proportion of those with more than 90% fill adherence was similar in the intervention and control groups (322 of 442 [72.9%] vs 327 of 452 [72.3%]; *P* = .87) (Table 2). Black patients had lower fill adherence than White patients (318 of 482 [66.0%] vs 331 of 412 [80.3%]; *P* < .001). In adjusted models, fill adherence increased significantly between baseline and 6 months in the control group (Table 3). Compared with baseline, pharmacy fill adherence did not differ between the intervention and control groups at 3 months (odds ratio [OR], 0.91 [95% CI, 0.57-1.43) or at 6 months (OR, 0.86 [95% CI, 0.53-1.38]). No significant treatment effects were seen by patient race (Black patients at 3 months: OR, 1.08 [95% CI, 0.61-1.92]; at 6 months: OR, 1.04 [95% CI, 0.58-1.87]; White patients at 3 months: OR, 0.68 [95% CI, 0.33-1.44]; at 6 months: OR, 0.55 [95% CI, 0.24-1.27]).

**Table 2.** Unadjusted Adherence and Blood Pressure at Enrollment Overall and by Patient Race

Characteristic	Patients, No. (%)			*P* value (Black vs White)^a^
	Overall	Black	White	
Pharmacy fill adherence (% with PDC >90%)				
Overall	649/894 (72.6)	318/482 (66.0)	331/412 (80.3)	<.001
Treatment group	322/442 (72.9)	152/232 (65.5)	170/210 (81.0)	
Control group	327/452 (72.3)	166/250 (66.4)	161/202 (79.7)	
*P* value (treatment group vs control group)	.87	.84	.75	
Self-reported adherence (% with adherence)				
Overall	574/942 (60.9)	287/523 (54.9)	287/419 (68.5)	<.001
Treatment group	284/468 (60.7)	142/254 (55.9)	142/214 (66.4)	
Control group	290/474 (61.2)	145/269 (53.9)	145/205 (70.7)	
*P* value (treatment group vs control group)	.88	.65	.34	
Pill count adherence, mean (SE) (range, 0-1.0)				
Overall	0.61 (0.31)	0.62 (0.31)	0.59 (0.31)	.17
Treatment group	0.59 (0.30)	0.59 (0.31)	0.59 (0.30)	
Control group	0.62 (0.32)	0.64 (0.30)	0.58 (0.33)	
*P* value (treatment group vs control group)	.34	.11	.73	
Systolic blood pressure, mean (SE), mm Hg				
Overall	139.1 (18.3)	140.6 (18.5)	137.3 (17.8)	.005
Treatment group	138.9 (17.9)	140.1 (18.3)	137.6 (17.4)	
Control group	139.3 (18.6)	141.1 (18.8)	136.9 (18.2)	
*P* value (treatment group vs control group)	.76	.51	.70	
Diastolic blood pressure, mean (SE), mm Hg				
Overall	82.0 (12.2)	83.9 (12.6)	79.7 (11.3)	<.001
Treatment group	81.6 (12.0)	82.8 (12.3)	80.1 (11.5)	
Control group	82.4 (12.4)	84.9 (12.7)	79.2 (11.2)	
*P* value (treatment group vs control group)	.31	.06	.37	

Abbreviation: PDC, proportion of days covered.

^a^ *P* values obtained from χ^2^ test (pharmacy fill and self-reported adherence) or *t* test (pill count adherence and blood pressures).

**Table 3.** Change in Adherence and Blood Pressure Overall, by Patient Race, and Comparing Treatment Effect by Patient Race^a^

Characteristic	Change from baseline to 3 mo	Change from baseline to 6 mo
Pharmacy fill, >90% PDC adherence, OR (95% CI)		
Overall		
Treatment group	1.10 (0.80-1.52)	1.39 (1.00-1.95)
Control group	1.22 (0.88-1.68)	1.63 (1.16-2.28)^b^
Treatment effect	0.91 (0.57-1.43)	0.86 (0.53-1.38)
Black patients		
Treatment group	1.26 (0.83-1.90)	1.36 (0.89-2.08)
Control group	1.17 (0.78-1.75)	1.31 (0.87-1.98)
Treatment effect	1.08 (0.61-1.92)	1.04 (0.58-1.87)
White patients		
Treatment group	0.88 (0.53-1.48)	1.46 (0.84-2.55)
Control group	1.29 (0.76-2.21)	2.67 (1.42-5.02)^b^
Treatment effect	0.68 (0.33-1.44)	0.55 (0.24-1.27)
Black patient treatment effect vs White patient treatment effect		
Treatment effect	1.58 (0.62-4.03)	1.90 (0.68-5.31)
Self-reported adherence, OR (95% CI)		
Overall		
Treatment group	1.46 (1.03-2.06)^b^	1.29 (0.92-1.80)
Control group	1.42 (1.01-1.99)^b^	1.24 (0.89-1.74)
Treatment effect	1.03 (0.63-1.67)	1.04 (0.65-1.67)
Black patients		
Treatment group	1.42 (0.88-2.27)	1.09 (0.70-1.70)
Control group	1.40 (0.89-2.19)	1.18 (0.76-1.82)
Treatment effect	1.02 (0.53-1.95)	0.93 (0.50-1.73)
White patients		
Treatment group	1.53 (0.92-2.54)	1.61 (0.96-2.69)
Control group	1.44 (0.85-2.44)	1.33 (0.79-2.25)
Treatment effect	1.06 (0.51-2.21)	1.20 (0.58-2.51)
Black patient treatment effect vs White patient treatment effect		
Treatment effect	0.95 (0.36-2.54)	0.77 (0.29-2.01)
Pill count, mean (SE) estimate		
Overall		
Treatment group	0.03 (0.02)	0.02 (0.02)
Control group	−0.05 (0.02)	−0.01 (0.02)
Treatment effect	0.08 (0.03)^b^	0.03 (0.03)
*P* value	.01	.31
Black patients		
Treatment group	0.04 (0.03)	0.05 (0.03)
Control group	−0.04 (0.03)	−0.01 (0.03)
Treatment effect	0.08 (0.04)	0.07 (0.04)
*P* value	.08	.15
White patients		
Treatment group	0.01 (0.03)	0.00 (0.03)
Control group	−0.05 (0.03)	0.00 (0.03)
Treatment effect	0.07 (0.04)	0.00 (0.05)
*P* value	.11	.96
Black patient treatment effect vs White patient treatment effect		
Treatment effect	0.01 (0.06)	0.07 (0.06)
*P* value	.87	.29
Systolic blood pressure, mean (SE) estimate, mm Hg		
Overall		
Treatment group	−2.69 (1.05)^b^	−2.98 (1.11)^b^
Control group	−2.44 (1.05)^b^	−4.33 (1.09)^b^
Treatment effect	−0.25 (1.48)	1.35 (1.56)
*P* value	.87	.39
Black patients		
Treatment group	−2.88 (1.48)^b^	−2.73 (1.54)
Control group	−4.42 (1.46)^b^	−3.93 (1.50)^b^
Treatment effect	1.54 (2.08)	1.21 (2.15)
*P* value	.46	.57
White patients		
Treatment group	−2.55 (1.50)	−3.27 (1.61)^b^
Control group	−0.37 (1.50)	−4.65 (1.59)^b^
Treatment effect	−2.18 (2.12)	1.38 (2.26)
*P* value	.30	.54
Black patient treatment effect vs White patient treatment effect		
Treatment effect	3.72 (2.97)	−0.17 (3.12)
*P* value	.21	.96
Diastolic blood pressure, mean (SE) estimate, mm Hg		
Overall		
Treatment group	−0.10 (0.64)	−1.03 (0.67)
Control group	−0.56 (0.63)	−1.69 (0.66)^b^
Treatment effect	0.45 (0.90)	0.65 (0.94)
*P* value	.61	.49
Black patients		
Treatment group	−1.21 (0.89)	−0.83 (0.93)
Control group	−2.10 (0.88)^b^	−2.44 (0.90)^b^
Treatment effect	0.89 (1.25)	1.61 (1.30)
*P* value	.48	.21
White patients		
Treatment group	0.95 (0.90)	−1.25 (0.97)
Control group	1.14 (0.91)	−0.74 (0.96)
Treatment effect	−0.19 (1.28)	−0.51 (1.36)
*P* value	.88	.71
Black patient treatment effect vs White patient treatment effect		
Treatment effect	1.09 (1.79)	2.12 (1.88)
*P* value	.54	.26

Abbreviations: OR, odds ratio; PDC, proportion of days covered.

^a^ In addition to the covariates of interest, models adjusted for study site, age, race, educational level, employment status, marriage status, insurance type, difficulty paying for basics, number of antihypertensive medications, heart failure, renal failure, and smoking status.

^b^ *P* ≤ .05.

#### Self-reported Adherence

Self-reported adherence was similar at baseline in the treatment and control groups (284 of 468 [60.7%] vs 290 of 474 [61.2%]; *P* = .88); Black patients reported lower adherence than White patients (287 of 523 [54.9%] vs 287 of 419 [68.5%]; *P* < .001) (Table 2). In adjusted models, self-reported adherence increased in the intervention and control groups between baseline and 3 months, with no differences between baseline and 6 months. No significant treatment effects for self-reported adherence were seen between intervention and control patients overall or by patient race (Table 3).

#### Pill Count Adherence

The mean (SE) baseline pill count adherence was similar in the intervention and control groups overall (0.59 [0.30] vs 0.62 [0.32]; *P* = .34) and by patient race (0.62 [0.31] for Black patients vs 0.59 [0.31] for White patients; *P* = .17) (Table 2). In adjusted models, the mean (SE) pill count adherence in the intervention group increased at 3 months (0.03 [0.02]) and 6 months (0.02 [0.02]) and decreased in the control group at 3 months (−0.05 [0.02]) and 6 months (−0.01 [0.02]) (Table 3). The change in pill count adherence at 3 months relative to baseline was significantly higher for intervention compared with control patients (mean [SE], 0.08 [0.03]; *P* = .01) but was not significantly higher at 6 months (Table 3).

Among Black patients, mean (SE) pill count adherence improved in the treatment group (0.04 [0.03] at 3 months and 0.05 [0.03] at 6 months) and decreased in the control group (−0.04 [0.03] at 3 months and −0.01 [0.03] at 6 months), but this treatment effect was not significant (Table 3). Among White patients, pill count adherence remained the same in the intervention group at both time points. The treatment effect was not significantly different in Black compared with White patients.

#### BP Outcomes

Mean (SE) overall baseline BP was similar in the intervention and control groups (systolic BP, 138.9 [17.9] and 139.3 [18.6] mm Hg; *P* = .76; diastolic BP, 81.6 [12.0] vs 82.4 [12.4] mm Hg; *P* = .31), but was higher among Black patients compared with White patients overall (systolic BP, 140.6 [18.5] vs 137.3 [17.8] mm Hg; *P* = .005; diastolic BP, 83.9 [12.6] vs 79.7 [11.3] mm Hg; *P* < .001) (Table 2). Systolic BP significantly decreased at 3 and 6 months for all patients irrespective of study group. No treatment effect on systolic or diastolic BP was noted overall or by patient race (Table 3).

### Patient Activation

Immediately after the intervention, patient activation was higher in the intervention patients than in control patients, but this difference was not statistically significant in an unadjusted comparison (median PAM score, 75.0 [IQR, 65.5-84.8] vs 72.5 [IQR, 63.1-80.9]; *P* = .06). In adjusted models, the Patient Activation Measure score immediately after the intervention was significantly higher in the intervention patients than in control patients (mean difference, 2.3 [95% CI, 0.1-4.5]). In adjusted models, PAM scores did not vary by study group at the 3-month or 6-month time points.

PAM scores overall were higher in Black patients than in White patients (PAM scores immediately after intervention: median, 75.0 [IQR, 65.5-84.8] vs 72.5 [IQR, 63.1-80.9]; *P* = .003). PAM scores did not vary by study group within patient race at any time point.

### Fidelity Analysis

At study conclusion, 13 writing exercises were missing. Of the 947 reviewed, 27 had no written text, 8 had illegible text, and 9 were protocol deviations; 97.7% (882 of 903) followed instructions by identifying at least one value on the list. In the intervention and control groups, 98.4% (443 of 450) and 14.1% (64 of 453), respectively, self-affirmed (eMethods 1 in Supplement 2).

White patients in the intervention group were more likely than Black patients in the intervention group to agree that these values strongly influenced their lives (191 of 207 [92.3%] vs 193 of 230 [83.9%]; *P* = .008), were values to live up to (198 of 207 [95.7%] vs 202 of 230 [87.8%]; *P* = .003), were an important part of who they are (199 of 207 [96.1%] vs 201 of 230 [87.4%]; *P* = .001), and were values that they cared about (198 of 206 [96.1%] vs 204 of 230 [88.7%]; *P* = .004). Similar results were noted in the control group.

## Discussion

In 960 patients with hypertension, a brief values affirmation intervention immediately before a clinic visit did not significantly improve medication adherence or BP at 3 or 6 months. No difference in the intervention effect was found between Black and White patients. Patient activation was higher in patients in the intervention group immediately following intervention delivery, with no difference in intervention effect on activation by patient race. Our findings suggest that a values affirmation intervention targeting racial stereotype threat did not reduce racial differences in medication adherence or BP by patient race.

The HYVALUE trial expands the literature examining interventions that target stereotype threat in health care. The threat of being subjected to negative stereotypes can impair memory or create anxiety among patients and lead them to forget or intentionally withhold important information, cause them to mistrust medical recommendations, or impair patient-clinician communication.^7,8,12,29,30,31^ Values affirmation targets this stereotype threat by encouraging patients to focus on important values outside the clinic setting, thereby reinforcing sources of self-worth and offsetting the potential threat of negative health information.^14,15,16^ Others have found that values affirmation interventions paired with health messaging (eg, reducing smoking) lead to significant improvement in self-reported health intentions and behaviors.^32,33^ The HYVALUE trial is one of the few studies testing the effect of values affirmation on objective clinical outcomes. A study of 256 Black patients with hypertension found that an education intervention enhanced with bimonthly positive affect induction and reminders for self-affirmation was associated with significantly higher medication adherence (assessed with electronic pill monitors) but no difference in BP compared with education alone.^9^ Potential reasons for differences in the prior study and the HYVALUE trial are that our intervention used only values affirmation, was delivered at a single time point, and used different adherence measures. Although we did not see improvement in clinical outcomes, our findings of higher patient activation among those who self-affirmed are similar to those of studies suggesting increased intention for health behaviors among patients who undergo a values affirmation intervention. Further research should examine the role of values affirmation on patient activation and subsequent clinical outcomes.

The HYVALUE trial failed to support our hypothesis that an intervention targeting stereotype threat reduces racial differences in medication adherence or BP. Core components of successful values affirmation interventions highlight potential reasons for a lack of an effect by patient race.^24^ First, the values used in the intervention may not have been as salient for Black patients as for White patients. We used the same values as in other studies targeting racial and ethnic disparities, yet Black patients less often agreed that these values were important. Second, the negative effects of stereotype threat may depend on setting.^34^ Racial stereotype threat may be lessened in established clinical relationships. Prior work has suggested that racial and ethnic bias may have less influence on outcomes in long-standing clinical relationships.^35^ Third, social context influences the effectiveness of values affirmation interventions.^34^ Many of our clinics served large proportions of Black patients (some up to 90%), and racial stereotypes may not be as relevant as in settings where Black patients are in the minority. Fourth, we did not examine vulnerability to stereotype threat. Individuals who identify strongly with their racial and ethnic group may be more conscious of negative stereotypes about their group and more susceptible to racial and ethnic stereotype threat.^29,36^ We cannot determine if the lack of treatment effect by patient race is due to a limitation of the study or a lack of a salient racial stereotype threat in this setting.

### Limitations

Our trial has some limitations. First, our population with hypertension represents only those who were contacted and agreed to enroll. Our study population may represent individuals who are particularly motivated to participate in a trial and adhere to medications. Participants tended to have more comorbidities but otherwise were clinically similar to those who were eligible but not enrolled (eTable in Supplement 2). Second, our measures of adherence may be insensitive to effects of our intervention. We found that patient activation was higher immediately after the intervention in those who received the intervention compared with those who did not. However, we did not find a relationship between treatment group and higher patient activation at the follow-up visits. Third, participants were required to have only 1 elevated BP measure in the prior 12 months for inclusion and our population had high rates of adherence at enrollment, which may not be generalizable to broader populations. Fourth, many sociodemographic characteristics of the participants differed yet were balanced within race and treatment group. Fifth, the HYVALUE trial did not measure patient-clinician communication. Values affirmation has been shown to improve communication between Black patients and their clinicians.^12^ The quality of the patient-clinician relationship, which includes communication, is associated with improved health outcomes and plays an important role in reducing health disparities.^37,38^

The HYVALUE trial and prior work suggest that a simple written intervention focused on values is associated with higher patient activation immediately after intervention delivery.^12^ Higher patient activation has been associated with improved knowledge and increased self-management behaviors (eg, regular exercise and taking medications).^39^ Therefore, our finding of higher patient activation among patients in the intervention group has implications for future work. Values affirmation is inexpensive, occurs outside of the patient-clinician interaction, and has the potential to be scaled for dissemination.^40,41,42^ Values affirmation targets the fear of negative stereotyping and is not specific to a disease or stereotyped group. Therefore, a scalable, pragmatic means to deliver values affirmation has the potential to improve patient-clinician communication and patient activation across a wide range of health care conditions and populations.

Finally, the HYVALUE trial tested only a patient-level intervention and did not address structural racism,^43,44,45,46,47,48^ which is produced and perpetuated by laws, rules, and practices embedded in the health care system.^49^ Processes in health care such as equating race and ethnicity with other physiological variables in clinical models that influence access to treatment and using White race or non-Hispanic ethnicity as the referent “norm” critically underlie health inequities.^47,48,49^ Values affirmation may equip patients with resources to overcome stereotype threat when visiting a physician’s office, but it does not address structural discrimination. Future research must explore practices and policies that perpetuate health disparities and multifaceted approaches to address them.^47,48^

## Conclusions

For 960 patients with hypertension, the HYVALUE trial found that a values affirmation intervention targeting stereotype threat did not improve medication adherence or BP. Patient activation was higher in patients in the intervention group, yet these differences did not influence the intervention’s effect. We found no significant treatment effect by patient race. Our findings suggest that an intervention targeting the negative influence of stereotype threat does not significantly reduce racial disparities in medication adherence or BP.

## Notes

Supplement 1.
Trial Protocol

Supplement 2. **eMethods 1.** Fidelity of Intervention **eMethods 2.** Clinician-Level Clustering **eTable.** Characteristics of the Eligible, Not Enrolled to the Enrolled Study Population

Supplement 3.
Data Sharing Statement

## References

[1] Benjamin EJ, Muntner P, Alonso A, ; American Heart Association Council on Epidemiology and Prevention Statistics Committee and Stroke Statistics Subcommittee. Heart disease and stroke statistics—2019 update: a report from the American Heart Association. Circulation. 2019;139(10):e56-e528. doi:10.1161/CIR.0000000000000659 30700139

[2] Lewis LM, Ogedegbe C, Ogedegbe G. Enhancing adherence of antihypertensive regimens in hypertensive African-Americans: current and future prospects. Expert Rev Cardiovasc Ther. 2012;10(11):1375-1380. doi:10.1586/erc.12.138 23244358 PMC4748723

[3] Kressin NR, Orner MB, Manze M, Glickman ME, Berlowitz D. Understanding contributors to racial disparities in blood pressure control. Circ Cardiovasc Qual Outcomes. 2010;3(2):173-180. doi:10.1161/CIRCOUTCOMES.109.860841 20233981 PMC2841788

[4] Bosworth HB, Powers B, Grubber JM, . Racial differences in blood pressure control: potential explanatory factors. J Gen Intern Med. 2008;23(5):692-698. doi:10.1007/s11606-008-0547-7 18288540 PMC2324164

[5] Ferdinand KC, Yadav K, Nasser SA, . Disparities in hypertension and cardiovascular disease in blacks: the critical role of medication adherence. J Clin Hypertens (Greenwich). 2017;19(10):1015-1024. doi:10.1111/jch.13089 28856834 PMC5638710

[6] Daugherty SL, Vupputuri S, Hanratty R, . Using values affirmation to reduce the effects of stereotype threat on hypertension disparities: protocol for the multicenter randomized Hypertension and Values (HYVALUE) trial. JMIR Res Protoc. 2019;8(3):e12498. doi:10.2196/12498 30907744 PMC6452278

[7] Burgess DJ, Warren J, Phelan S, Dovidio J, van Ryn M. Stereotype threat and health disparities: what medical educators and future physicians need to know. J Gen Intern Med. 2010;25(suppl 2):S169-S177. doi:10.1007/s11606-009-1221-4 20352514 PMC2847106

[8] Abdou CM, Fingerhut AW, Jackson JS, Wheaton F. Healthcare stereotype threat in older adults in the Health and Retirement Study. Am J Prev Med. 2016;50(2):191-198. doi:10.1016/j.amepre.2015.07.034 26497263 PMC4755717

[9] Ogedegbe GO, Boutin-Foster C, Wells MT, . A randomized controlled trial of positive-affect intervention and medication adherence in hypertensive African Americans. Arch Intern Med. 2012;172(4):322-326. doi:10.1001/archinternmed.2011.1307 22269592 PMC4669680

[10] Cohen GL, Garcia J, Apfel N, Master A. Reducing the racial achievement gap: a social-psychological intervention. Science. 2006;313(5791):1307-1310. doi:10.1126/science.1128317 16946074

[11] Cohen GL, Garcia J, Purdie-Vaughns V, Apfel N, Brzustoski P. Recursive processes in self-affirmation: intervening to close the minority achievement gap. Science. 2009;324(5925):400-403. doi:10.1126/science.1170769 19372432

[12] Havranek EP, Hanratty R, Tate C, . The effect of values affirmation on race-discordant patient-provider communication. Arch Intern Med. 2012;172(21):1662-1667. doi:10.1001/2013.jamainternmed.258 23128568

[13] Cohen GL, Sherman DK. The psychology of change: self-affirmation and social psychological intervention. Annu Rev Psychol. 2014;65:333-371. doi:10.1146/annurev-psych-010213-115137 24405362

[14] Sherman DK, Cohen GL. The psychology of self-defense: self-affirmation theory. In: Mark PZ, ed. Advances in Experimental Social Psychology. Vol 38. Academic Press; 2006:183-242.

[15] Shnabel N, Purdie-Vaughns V, Cook JE, Garcia J, Cohen GL. Demystifying values-affirmation interventions: writing about social belonging is a key to buffering against identity threat. Pers Soc Psychol Bull. 2013;39(5):663-676. doi:10.1177/0146167213480816 23478675

[16] Steele CM. The psychology of self-affirmation: sustaining the integrity of the self. In: Berkowitz L, ed. Advances in Experimental Social Psychology. Vol 21. Academic Press; 1988:261-302.

[17] Boutron I, Moher D, Altman DG, Schulz KF, Ravaud P; CONSORT Group. Extending the CONSORT statement to randomized trials of nonpharmacologic treatment: explanation and elaboration. Ann Intern Med. 2008;148(4):295-309. doi:10.7326/0003-4819-148-4-200802190-00008 18283207

[18] Magid DJ, Olson KL, Billups SJ, Wagner NM, Lyons EE, Kroner BAA. A pharmacist-led, American Heart Association Heart360 Web-enabled home blood pressure monitoring program. Circ Cardiovasc Qual Outcomes. 2013;6(2):157-163. doi:10.1161/CIRCOUTCOMES.112.968172 23463811

[19] James PA, Oparil S, Carter BL, . 2014 Evidence-based guideline for the management of high blood pressure in adults: report from the panel members appointed to the Eighth Joint National Committee (JNC 8). JAMA. 2014;311(5):507-520. doi:10.1001/jama.2013.284427 24352797

[20] Steiner JF, Koepsell TD, Fihn SD, Inui TS. A general method of compliance assessment using centralized pharmacy records: description and validation. Med Care. 1988;26(8):814-823. doi:10.1097/00005650-198808000-00007 3398608

[21] Voils CI, Maciejewski ML, Hoyle RH, . Initial validation of a self-report measure of the extent of and reasons for medication nonadherence. Med Care. 2012;50(12):1013-1019. doi:10.1097/MLR.0b013e318269e121 22922431 PMC3494794

[22] Hansen RA, Kim MM, Song L, Tu W, Wu J, Murray MD. Comparison of methods to assess medication adherence and classify nonadherence. Ann Pharmacother. 2009;43(3):413-422. doi:10.1345/aph.1L496 19261962

[23] Hibbard JH, Mahoney ER, Stockard J, Tusler M. Development and testing of a short form of the patient activation measure. Health Serv Res. 2005;40(6, pt 1):1918-1930. doi:10.1111/j.1475-6773.2005.00438.x 16336556 PMC1361231

[24] Bradley DN, Crawford EP, Dahill-Brown SE. Defining and assessing FoI in a large-scale randomized trial: core components of values affirmation. Stud Educ Eval. 2016;49:51-65. doi:10.1016/j.stueduc.2016.04.002

[25] Lim MT, Ab Rahman N, Teh XR, . Optimal cut-off points for adherence measure among patients with type 2 diabetes in primary care clinics: a retrospective analysis. Ther Adv Chronic Dis. 2021;12:2040622321990264. doi:10.1177/2040622321990264 33643600 PMC7894582

[26] Molenberghs G, Kenward MG, Verbeke G, Birhanu T. Pseudo-likelihood estimation for incomplete data. Statistica Sinica. 2011;12(1):187-206.

[27] Diggle P, Kenward MG. Informative drop-out in longitudinal data analysis. Appl Statist. 1994;43(1):49-93. doi:10.2307/2986113

[28] Dempster AP, Laird NM, Rubin DB. Maximum likelihood from incomplete data via the EM algorithm. J R Stat Soc Ser B Methodological. 1977;39(1):1-38.

[29] Abdou CM, Fingerhut AW. Stereotype threat among Black and White women in health care settings. Cultur Divers Ethnic Minor Psychol. 2014;20(3):316-323. doi:10.1037/a0036946 25045944 PMC5449200

[30] Aronson J, Burgess D, Phelan SM, Juarez L. Unhealthy interactions: the role of stereotype threat in health disparities. Am J Public Health. 2013;103(1):50-56. doi:10.2105/AJPH.2012.300828 23153125 PMC3518353

[31] Blascovich J, Spencer SJ, Quinn D, Steele C. African Americans and high blood pressure: the role of stereotype threat. Psychol Sci. 2001;12(3):225-229. doi:10.1111/1467-9280.00340 11437305

[32] Sweeney AM, Moyer A. Self-affirmation and responses to health messages: a meta-analysis on intentions and behavior. Health Psychol. 2015;34(2):149-159. doi:10.1037/hea0000110 25089345

[33] Peterson JC, Czajkowski S, Charlson ME, . Translating basic behavioral and social science research to clinical application: the EVOLVE mixed methods approach. J Consult Clin Psychol. 2013;81(2):217-230. doi:10.1037/a0029909 22963594 PMC3578179

[34] Borman GD. Advancing values affirmation as a scalable strategy for mitigating identity threats and narrowing national achievement gaps. Proc Natl Acad Sci U S A. 2017;114(29):7486-7488. doi:10.1073/pnas.1708813114 28696322 PMC5530710

[35] Blair IV, Steiner JF, Hanratty R, . An investigation of associations between clinicians’ ethnic or racial bias and hypertension treatment, medication adherence and blood pressure control. J Gen Intern Med. 2014;29(7):987-995. doi:10.1007/s11606-014-2795-z 24549521 PMC4061371

[36] Smith LV, Cokley K. Stereotype threat vulnerability: a psychometric investigation of the Social Identities and Attitudes Scale. Meas Eval Counseling Dev. 2016;49(2):145-162. doi:10.1177/0748175615625752

[37] Kelley JM, Kraft-Todd G, Schapira L, Kossowsky J, Riess H. The influence of the patient-clinician relationship on healthcare outcomes: a systematic review and meta-analysis of randomized controlled trials. PLoS One. 2014;9(4):e94207. doi:10.1371/journal.pone.0094207 24718585 PMC3981763

[38] Kamimura A, Higham R, Rathi N, Panahi S, Lee E, Ashby J. Patient-provider relationships among vulnerable patients: the association with health literacy, continuity of care, and self-rated health. J Patient Exp. 2020;7(6):1450-1457. doi:10.1177/2374373519895680 33457601 PMC7786733

[39] Newland P, Lorenz R, Oliver BJ. Patient activation in adults with chronic conditions: a systematic review. J Health Psychol. 2021;26(1):103-114. doi:10.1177/1359105320947790 32830587

[40] Springer A, Venkatakrishnan A, Mohan S, Nelson L, Silva M, Pirolli P. Leveraging self-affirmation to improve behavior change: a mobile health app experiment. JMIR Mhealth Uhealth. 2018;6(7):e157. doi:10.2196/mhealth.9151 30026179 PMC6072974

[41] Taber JM, Klein WM, Ferrer RA, Augustson E, Patrick H. A pilot test of self-affirmations to promote smoking cessation in a national smoking cessation text messaging program. JMIR Mhealth Uhealth. 2016;4(2):e71. doi:10.2196/mhealth.5635 27278108 PMC4917724

[42] Brown SD, Fotuhi O, Grijalva CS, . A randomized study of values affirmation to promote interest in diabetes prevention among women with a history of gestational diabetes. Med Care. 2019;57(7):528-535. doi:10.1097/MLR.0000000000001133 31107396 PMC6565448

[43] Whelton PK, Einhorn PT, Muntner P, ; National Heart, Lung, and Blood Institute Working Group on Research Needs to Improve Hypertension Treatment and Control in African Americans. Research needs to improve hypertension treatment and control in African Americans. Hypertension. 2016;68(5):1066-1072. doi:10.1161/HYPERTENSIONAHA.116.07905 27620388 PMC5063700

[44] Mueller M, Purnell TS, Mensah GA, Cooper LA. Reducing racial and ethnic disparities in hypertension prevention and control: what will it take to translate research into practice and policy? Am J Hypertens. 2015;28(6):699-716. doi:10.1093/ajh/hpu233 25498998 PMC4447820

[45] Bartolome RE, Chen A, Handler J, Platt ST, Gould B. Population care management and team-based approach to reduce racial disparities among African Americans/Blacks with hypertension. Perm J. 2016;20(1):53-59. doi:10.7812/TPP/15-052 26824963 PMC4732795

[46] Mensah GA, Cooper RS, Siega-Riz AM, . Reducing cardiovascular disparities through community-engaged implementation research: a National Heart, Lung, and Blood Institute workshop report. Circ Res. 2018;122(2):213-230. doi:10.1161/CIRCRESAHA.117.312243 29348251 PMC5777283

[47] Boyd RW, Lindo EG, Weeks LD, McLemore MR. On racism: a new standard for publishing on racial health inequities. Health Affairs Blog. Published July 2, 2020. Accessed March 18, 2021. https://www.healthaffairs.org/do/10.1377/hblog20200630.939347/full/

[48] Feagin J, Bennefield Z. Systemic racism and U.S. health care. Soc Sci Med. 2014;103:7-14. doi:10.1016/j.socscimed.2013.09.006 24507906

[49] Bailey ZD, Feldman JM, Bassett MT. How structural racism works—racist policies as a root cause of U.S. racial health inequities. N Engl J Med. 2021;384(8):768-773. doi:10.1056/NEJMms2025396 33326717 PMC11393777

