# Supplementary material for: Effect of Values Affirmation on Reducing Racial Differences in Adherence to Hypertension Medication: The HYVALUE Randomized Clinical Trial
Source: JAMA Netw Open. 2021 Dec 16;4(12):e2139533. doi: 10.1001/jamanetworkopen.2021.39533 (PMC8678693; doi:10.1001/jamanetworkopen.2021.39533)
Supplement: Supplement 1. — Trial Protocol [file jamanetwopen-e2139533-s001.pdf]

Formatted: Numbering: Continuous

COMIRB Protocol

COLORADO MULTIPLE INSTITUTIONAL REVIEW BOARD
CAMPUS BOX F-490 TELEPHONE: 303-724-1055 Fax: 303-724-0990

Protocol #: 16-0510

Project Title: Values and Hypertension Study
Principal Investigator: Stacie L. Daugherty
Version Date: 12-05-20

Hypotheses and Specific Aims: The objective of the study is to reduce the effects of stereotype threat on the adherence of African American patients with hypertension. We hypothesize that stereotype threat impairs communication between minority patients and their providers resulting in decreased patient engagement and lower rates of adherence with antihypertensive medication. .We propose an innovative approach using values affirmation to overcome the stereotype threat to increase patient activation, improve adherence and thereby reduce disparities in hypertension outcomes. Our proposal is based upon our pilot study demonstrating a brief values affirmation exercise done immediately before a clinic visit resulted in improved communication between African American patients and providers during the visit and suggested improved medication adherence 6-months after the visit. We propose to advance our prior work and fill gaps in the evidence by conducting a randomized controlled trial comparing a values affirmation exercise to a control exercise among 1,130 patients with hypertension from three health systems. The primary outcome is 6-month adherence to antihypertensive therapies with the secondary outcomes of blood pressure, time under blood pressure control, and treatment intensification. We will also explore possible mechanisms of the intervention effect and formally examine the potential for widespread dissemination.

The specific aims are to:

Aim 1. Compare the effects of the values-affirmation exercise to a control condition on antihypertensive medication adherence in African American patients with uncontrolled hypertension across three clinical settings.

> **H1:** Those performing the values-affirmation exercise will have increased antihypertensive medication adherence over the ensuing 6-months compared to the control condition
> **H2:** The effect of values affirmation will be partially moderated by patient factors (prior experience with discrimination),and provider factors (implicit racial bias, past disparities training, self-efficacy for caring for minority patients), and mediated by the theory-driven factors of patient activation, attitudes, perceived social norms, perceived behavioral control and anticipated emotion regarding management of blood pressure.

Aim 2. Compare the effects of the values-affirmation exercise in African American patients and white patients with uncontrolled hypertension and similar socioeconomic characteristics.

> **H3:** Values-affirmation will improve antihypertensive medication adherence to a greater degree in African American patients than whites, consistent with the hypothesis that values affirmation is targeting stereotype threat related to race.

Aim 3. Evaluate the intervention for widespread dissemination using the RE-AIM (reach, effectiveness, adoption, implementation and maintenance) framework.

> **H4**: The intervention will be suitable for more widespread dissemination within primary care.

II. Background and Significance:

1.0 Significance

1.1 African Americans have a higher prevalence of uncontrolled hypertension than white Americans leading to disparities in cardiovascular outcomes. One in 3 US adults has hypertension and African Americans are disproportionally affected; almost 40% of non-Hispanic blacks have hypertension.[1, 2] Although the rates of uncontrolled hypertension have been decreasing in all groups, African Americans continue to have higher rates of uncontrolled hypertension compared to white Americans.[3] Uncontrolled hypertension disproportionately affects outcomes in African Americans compared to whites. A 10-mm Hg difference in systolic blood pressure (SBP) is associated with an 8% increase in stroke risk for whites, but a 24% increase in risk for African Americans.[4] Lower adherence to treatment recommendations may contribute to higher rates of uncontrolled hypertension among African Americans.[5] Therefore, interventions to improve adherence have the potential to reduce racial disparities in hypertension outcomes.

1.2 **The Institute of Medicine, World Health Organization and others have identified poor adherence to medications as the most significant, modifiable contributor to uncontrolled hypertension**.[6-11] The prevalence of poor adherence to antihypertensive medications ranges from 43% to 78%, with approximately 50% of hypertensive patients discontinuing the use of their medications after 1 year.[11, 12] Poor adherence to antihypertensive medications is associated with poor outcomes and improving adherence reduces BP.[9-11, 13-16] Adherence rates are lower in African Americans than whites with hypertension, and non-adherence has been shown to contribute to racial differences in hypertension control.[7, 17-20] Therefore, interventions to improve adherence have the potential to reduce racial disparities in hypertension outcomes.

1.3 Stereotype threat may contribute to low adherence. Stereotype threat occurs when cues in the environment (such as visiting a doctor's office) trigger the threat of confirming, as self-characteristic, a negative stereotype about one's group.[21] Although any individual may experience stereotype threat, African Americans are at greater risk due to widespread racism and past experiences of discrimination.[22-31 32-37] In the clinical setting, African American patients report stereotype threat related to being viewed as unintelligent, "second class citizens", and unworthy of good care.[21, 36, 38-40] Stereotype threat triggers psychological and physiological responses including reduced memory capacity, impaired communication, disengagement and reduced motivation.[41, 42] Stereotype threat may thus contribute to racial disparities in non-adherence to treatment by hindering a patient's ability to process information and actively engage in a discussion about their health during a clinical encounter.[21 43] Based on this poor clinical interaction, the patient may feel less activated to adhere to treatment recommendations.[21, 44] Therefore, interventions targeting stereotype threat have the potential to reduce disparities among populations that experience widespread discrimination. (Figure 1)

Figure 1. Theoretical Model

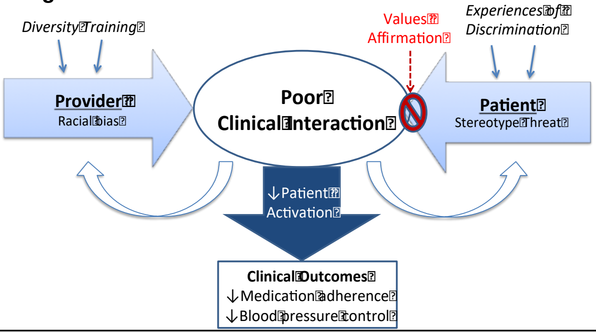

1.4 Values affirmation interventions reduce stereotype threat and decrease racial disparities in academic performance.[45] Values affirmation exercises typically ask participants to write a few sentences about their core values.[45] By focusing on values that are important to them, such as friends and family, religion, or art, values affirmation bolsters the person's self-concept by helping them view themselves as adequate, effective, and able to control important outcomes in spite of a possible threat.[46-48] Values affirmation inventions have been associated with reduced stress and improved academic performance among stigmatized group members.[49-53] Specifically, Cohen et al (consultant on this proposal) demonstrated participation in an affirmation exercise at the beginning of an academic term reduced racial achievement gaps among African American children by 40%; these effects were sustained two years later.[49, 50] Miyake and colleagues performed a similar study

with college students in an introductory physics course, and found that values affirmation significantly diminished the gender gap in grades over a semester.[51] Walton and Cohen used a related intervention among white and African American college freshman and found the racial gap in grade point average closed by approximately half over three years of follow-up.[54] Importantly, African American students in the intervention group reported better overall health and fewer doctor visits up to 3 years after the intervention. Overall, this literature provides strong support that a brief psychological intervention targeting stereotype threat can have large and sustained effects on racial disparities in outcomes..[55]

1.5 We hypothesize that a values affirmation intervention will reduce racial disparities in adherence. Based on the evidence supporting the effectiveness of values affirmation in educational settings, we hypothesize that values affirmation can similarly reduce racial disparities in adherence and subsequent health outcomes. Our proposed project grows from a theoretical model suggesting patient adherence to prescribed therapies and subsequent control of hypertension are dependent upon the quality of the patient-provider interaction and resultant patient activation.[56] (Figure 1)

The patient-provider interaction provides an opportunity for information exchange and the quality of the interaction is associated with patient adherence to prescribed therapies.[57-59] Our group and others have shown that healthcare providers have implicit racial biases that are associated with poor communication.[60, 61] From the patient perspective, these subtle experiences of bias can result in stereotype threat. Such threat increases anxiety, reduces memory, and may impair the patient's communication and ability to absorb information.[21] For example, an African American patient, who is experiencing stereotype threat, may alter their behavior during a clinical interaction by failing to ask questions or engage in a discussion about their blood pressure, or to anticipate barriers to adherence. The resulting poor interaction may increase stereotype threat and, a self-fulfilling feedback loop, reinforce a provider's perception that the patient is disengaged or unintelligent. Due to this negative, self-perpetuating cycle, stereotype threat may alter both patient and provider perceptions and decision-making, and, over time, contribute to health disparities.[62] Importantly, stereotype threat may occur in the absence of actual bias in that specific situation, since reminders of prior experiences of bias can trigger it.[21] Therefore, intervening on stereotype threat might lead to independent effects beyond interventions directed at providers. (Figure 1)

The patient-provider interaction is only the first step in care of a chronic condition. Most of the real work of hypertension control takes place away from the clinic, in the form of self-care. Patients must make appointments, maintain prescriptions, take medications, and follow dietary and exercise plans. Because the patient-provider interaction initiates patient activation with self-care, an interaction distorted by stereotype threat can cause significant and lasting damage to hypertension care.[63] Increased patient activation is associated with increased adherence.[64, 65] We hypothesize that values affirmation, by enhancing the patients' resources for overcoming stereotype threat, improves patient-provider communication leading to improved patient activation and adherence.

1.6 Targeting provider racial bias is another potential approach to improving patient-provider interactions; however, intervening upon provider racial bias presents numerous challenges. Provider racial bias and discrimination contributes to poor communication and disparities.[66] We will measure racial bias as a potential moderator of the intervention's effect; however, directly intervening upon these processes goes beyond the scope of this study in several ways. First, the provider processes that contribute to disparities are complex and likely implicit, or out of providers' conscious control, making them very difficult to intervene upon.[67] Although implicit attitudes have been malleable in experimental settings, comparable methods have not been tested in a clinical setting with relevant outcomes.[68, 69] Second, our group and others have demonstrated that implicit racial bias is common among health care providers.[70-72] Changing attitudes throughout a healthcare system is an ambitious and perhaps unrealistic goal. Third, interventions targeting bias among providers have typically relied upon diversity or cultural competency training; however, the evidence for the effectiveness of cultural competency training on reducing health disparities has been modest.[73, 74] Cultural training standards often include approaches like stereotype suppression that have not been effective for reducing implicit bias.[75, 76] Despite these limitations, we recognize

the potential benefit of cultural competency training programs and we will assess exposure to past training as a potential moderator of the intervention's effect. Finally, any program that requires substantial time commitments from clinicians has a lower likelihood of being widely adopted or implemented.[77] Therefore, we have chosen to focus on a low-resource intervention to assist patients that could be easily integrated into routine clinical practice and has been shown to be effective in field settings including health care.

1.7 Our pilot work suggests values affirmation improves patient engagement and adherence. This investigative team tested the theoretical model in a pilot study that informed the current proposal. At a single clinical site, 151 African Americans patients with hypertension were randomized to perform either a values-affirmation exercise or a control exercise immediately before a scheduled visit with their primary care physicians (all who were white). In a subset of 99 patients, patient-provider communication was assessed by analyzing audio recordings with the Roter Interaction Analysis System.[78] This system codes each speech segment based on its content and delivery, and provides global ratings of the emotional tone of the participants' speech. Finally, pharmacy records were used to compare adherence in the six-month period before the index visit with the six-months after the index visit. The intervention had benefit in two areas:

- Patients in the affirmation intervention exchanged more information about their medical conditions, and their encounters were characterized as more engaged than patients in the control condition.[79]

	Intervention	Control	P
Mean number of utterances about medical condition (SE)	66.3 (6.8)	48.1 (5.9)	0.03
Mean rating of patients' emotional tone (SE)			
Interested, friendly, responsive, interactive, respectful	5.1 (0.1)	4.8 (0.1)	0.02
Depressed and distressed	1.1 (0.2)	1.5 (0.1)	0.03

- Patients in the affirmation intervention showed improved medication adherence (mean percentage of days covered for a composite of antihypertensive medications) in the 6 months after the index visit, whereas control patients did not (unpublished data, manuscript under review).

	Before Visit	After Visit	P*
Intervention mean adherence (SD)	74% (22)	83% (14)	0.01
Control mean adherence (SD)	83% (18)	81% (17)	0.45

* for the time x adherence interaction term, p = 0.02

Mediational analysis suggested that *improved adherence was mediated by an increase in information exchange during the visit.* These findings support the concept that values affirmation in the clinical setting can increase patient engagement in health care. (Figure 1)

1.7 While we have enthusiasm for the results of the pilot work, knowledge gaps remain that will be addressed in the proposed study. The pilot study was carried out at a single site and only among African Americans. The possibility exists that the threat being blunted by the values affirmation exercise is the more general threat of illness rather than stereotype threat related to race, and thus the exercise could benefit all patients (whites included) and not just African Americans. Since stereotype threat is not readily measured directly, it must be inferred from differential effects of manipulation between groups. Further, possible patient (past discrimination) and provider factors (racial attitudes) that may moderate the effects have not been examined. Finally, the feasibility of wide dissemination of the intervention has not been formally evaluated. Thus, it is premature to consider the values affirmation a general means to address the contribution of stereotype threat to health disparities. To address the limitations of the pilot study, we propose to expand the investigation to 9 clinical sites across multiple systems, compare the effects in African Americans and white Americans, explore possible moderators (i.e. past discrimination, provider racial attitudes) and mediators (i.e. patient activation), and formally evaluate dissemination.

2.0 Innovation. Our proposal is innovative for the following reasons:

- **New approach**: We propose a unique methodology that has been widely successful at reducing racial disparities in other fields.[48-52] To our knowledge **values affirmation** has not been extensively evaluated as a health disparities intervention.
- **Sound theoretical concept –** The theoretical model (Figure 1) for which this proposal is based is supported by patient engagement theory.[56] Our pilot study demonstrated that a single values-affirmation exercise improved subsequent patient-provider interaction and adherence, further supporting the validity of our theoretical approach.[79]
- **Pragmatic**: Compared to existing interventions to improve adherence, values affirmation is significantly less time and resource intensive, enhancing the potential for the intervention to be embedded in primary care.[80]
- **Evaluate for dissemination**: Using the RE-AIM (reach, effectiveness, adoption, implementation, maintenance) framework, we will formally evaluate the intervention for more widespread dissemination.[81-83] Therefore, our approach will facilitate moving the intervention from a clinical trial to subsequent dissemination and implementation into clinical care.
- **Widely applicable**: Stereotype threat is not specific to hypertension outcomes or African Americans. Health care disparities have been demonstrated across numerous disease states and minority groups.[66] Therefore, a simple intervention targeting a common mechanism has the potential to significantly reduce a wide range of health disparities.

III. Preliminary Studies/Progress Report:

See section 1.6 for description of pilot studies.

IV. Research Methods

Overall strategy. We will conduct a randomized controlled trial in African American and white patients with uncontrolled hypertension, comparing a values affirmation intervention to a control exercise in three healthcare systems. The primary outcome will be change in medication adherence with secondary outcomes of change in blood pressure, time under blood pressure control and treatment intensification. We will explore the mechanistic effects of the intervention and assess the suitability of the intervention for more widespread dissemination.

A. Outcome Measure(s): The primary outcome measure is medication adherence comparing adherence before and after the intervention. The secondary outcome measures are systolic blood pressure (comparing blood pressure at the index visit to blood pressure at 6-month follow-up), time under blood pressure control and treatment intensification.

Primary Outcome: Medication adherence. We plan to measure medication adherence in three ways. First, we will assess adherence using electronic pharmacy records as in our pilot study. Computerized pharmacy records have been used in a wide array of studies to assess medication adherence and correlate with other adherence measures such as electronic adherence monitors, self-report, and pill counts, and with treatment outcomes such as BP control.[84-89] The measure of interest for the proposed study will be a summary measure of adherence which assesses the proportion of days covered (PDC) over the period of observation for which a patient obtains antihypertensive medications.[84] Adherence will be calculated for each antihypertensive drug in the regimen and combined across drugs into a summary measure of adherence for the entire drug regimen using the method developed by Steiner et al.[84] We will calculate prior adherence using data from 12 months prior to the index visit to calculate change in adherence compared with the six months after the index visit. The investigator team has used this method to measure adherence in prior studies.[20, 59, 90-97]

Second, because some patients will have only one refill opportunity (90-day supplies) over the 6-month follow-up period, we will also collect self-reported adherence at baseline, 3 and 6-months using the validated Voils instrument.[92, 98, 99] The measure has three questions that address the *extent* of non-adherence over the previous 7 days.

Finally, we will validate the pharmacy and self-reported measures of adherence as assessed by pill counts. If *x* is the number of pills in the bottle, *y* is the number of pills that would have been in the bottle had all pills been taken since the bottle was filled, and *z* is the number of pills that should have been taken since the last fill, adherence is calculated as 1- [$(x-y)/z$].

Secondary Outcomes:

Blood pressure. The research assistant will measure baseline, 3- and 6-month blood pressures according to guideline recommendations.[100] To account for possible loss to follow-up, we will secondarily use the last blood pressure recorded in the EHR during the six-month post-enrollment period.

Time Under Control – will be defined as the proportion of time over the 12-months of follow-up with a BP ≤ 140/90 mmHg.[101] Of note, BP control definitions will be modified if hypertension guidelines change during the study period. All BPs obtained during non-urgent clinic visits over the follow-up period will be included.

Treatment Intensification – Appropriate treatment intensification is another significant contributor to uncontrolled BP.[20, 90, 102-105]Using a standard scoring method, appropriate treatment intensification will be calculated by subtracting the number of expected intensifications (number of visits after enrollment with a BP ≥140/90 mm Hg) from the number of observed intensifications (either an increase in dose or addition of a new medication class), and then dividing this difference by the number of office visits over the 12 months.[106] Our group has previously measured treatment intensification using this approach.[90, 107]

B. Description of Population to be Enrolled:

<u>Study setting</u>. To maximize the external validity of the proposed study, the project will be conducted within three large healthcare systems that care for diverse populations. These sites incorporate three states (Colorado, Maryland and District of Columbia) and two socio-economic groups (poor/uninsured and working/insured), and have sufficient minority patient populations to ensure that enrollment needs will be met.

Denver Health (DH) is an integrated safety net system serving the city of Denver. It consists of a 477-bed hospital and 9 community-based primary care clinics.[108, 109] The proposed study will be conducted at the Eastside, Park Hill, Webb, Lowry, Westside, and Level One Physicians clinics. Approximately 40 clinicians (physicians and advanced care providers) provide primary care at these clinics. In 2014, these clinics saw 23,831 patients in 64,757 outpatient visits. In these clinics, 8400 patients have hypertension, of which 37% are African American and 27% are white. Based on these data and our experience enrolling ~140 patients during our pilot study at a single DH clinic over 12 months, recruiting the targeted study sample is feasible.

Kaiser Permanente of Colorado (KPCO). KPCO is a not-for-profit integrated delivery system serving over 670,000 members at 22 outpatient medical clinics across the Colorado Front Range. Participants will be recruited from three KPCO clinics in order to assure adequate numbers of African American patients: East Denver, Aurora Centrepoint, and Skyline clinics. In the first 2 quarters of 2015, these three clinics saw 71,582 patients; 12% were African American and 46% were white. Approximately 100 physicians and advanced care practitioners provide primary care at these clinics. In a recent hypertension study at KPCO there were 6,393 members with new prescriptions for antihypertensive medications over an 18-month period.[110] A randomized trial of uncontrolled hypertension patients at KPCO enrolled 348 patients over 14-months.[111] Based on these data, recruiting the targeted study sample is feasible.

Kaiser Permanente of Mid-Atlantic States (KPMAS). KPMAS is a not-for-profit integrated delivery system serving over 600,000 members in Maryland, District of Columbia and Northern Virginia. KPMAS has 32 medical centers across the service areas providing outpatient clinical care, as well as pharmacy and laboratory services. During the past year, 123,988 adult hypertensive patients, with at least one primary care visit, and continuous enrollment were identified at KPMAS. Among these hypertension patients, 33% were white and 48% African American. Recruitment will take place at 3 KPMAS medical centers in the Maryland and DC area which have large numbers of hypertensive patients with relatively balanced distributions by race: Capitol Hill, Kensington, and Gaithersburg Medical Centers. Our preliminary data indicates more than adequate population samples for successful recruitment at KPMAS.

The collaboration structure. All three sites have extensive experience with practice-based research and multicenter studies. DH and KPCO investigators have long-standing, ongoing collaborations, as have the KPCO and KPMAS investigators.[20, 60, 67, 71, 79, 107, 112-115] Based on these experiences, we are confident the group can carry out the proposed work effectively. This multicenter collaboration will be further enhanced by a formal plan for training and supervision to ensure that enrollment targets are met, that study procedures are carried out with fidelity, and that data quality is addressed regularly. (see Data management plan)

<u>Patient eligibility</u>. Patients with a diagnosis of hypertension who have an upcoming clinic visit with their PCP will be screened on a weekly basis for inclusion/exclusion criteria using the electronic health record (EHR). Diagnosed hypertension will be defined as having an outpatient visit in the past 2 years with a primary or secondary ICD-9 code diagnosis of hypertension. This method of identifying patients with diagnosed hypertension has been shown to have a sensitivity of 81.4% and a specificity of 84.4% in a study done at DH; similar accuracy has been shown for Kaiser Permanente.[116] Patients will be eligible if they have evidence for uncontrolled BP (≥140/90 mm Hg) at least once at any point over the preceding 12-months.[111] We have chosen a broad definition of uncontrolled BP since over 25% of patients with previously controlled blood pressure are no longer controlled over the following year.[117, 118] Recidivism to uncontrolled BP is associated with non-adherence.[117, 118] Additional inclusion criteria are age > 21 years, self-described race/ethnicity of (non-Hispanic) African American or non-Hispanic white, the ability to read and write English (self-reported), and the ability to provide informed consent. Patients with pregnancy-related hypertension or hypertension due to dialysis-dependent end-stage renal disease will be excluded as not being representative of the broader population of patients with chronic hypertension. Since pharmacy records are used for the primary measure of adherence, we will include those who obtain medications from DH, KPCO, or KPMAS pharmacies. Originally, we planned to exclude those who do not obtain medications from their home research site pharmacy. However, this has thus far prevented us from enrolling any white patients at our Denver Health site. Among those patients who do not obtain medications from their home research site pharmacies, we will seek consent to obtain medication fill data from their pharmacy of choice. At present, this constitutes nearly all patients seen at the study sites. We are aware that changes may occur as patients' benefit plans change. Using the electronic health record (EHR) at each site, we will be able to compare study patients with all hypertension patients to assess if this enrollment criterion has introduced bias.

Each site already uses its EHR to identify and track patients with hypertension for quality improvement purposes. The available data includes clinical characteristics (e.g. vital signs), demographics (including self-reported race, ethnicity, and language preference data), diagnoses, outpatient and hospital utilization, laboratory and imaging procedures, pharmacy records, death certificate data, and membership enrollment.

Patient recruitment and procedures. Subjects meeting inclusion/exclusion criteria will be contacted via mail, email, and/or phone prior to their scheduled appointment with

a PCP. Potential participants will be told the research is being done to assess adherence to hypertensive medications and to evaluate how a writing exercise about values might affect blood pressure. If they express interest in the study, they will be asked to arrive approximately one hour prior to the visit in order to review study procedures and provide informed consent. (see Patient Informed Consent) Those who decide to participate will have their blood pressure measured by the research coordinator using guideline-recommended procedures.[100] Next, participants will be given a pre-randomized, consecutively numbered packet – to ensure the research coordinator is blind to the condition - containing the study materials. Packets will contain either an intervention or control version of the values affirmation exercise. To ensure balanced randomization by race at each site, there will be a pre-randomized series of packets for participants who self-identify as white and another pre-randomized series for those who self-identify as African American. Additional surveys will include a baseline demographics questionnaire, a measure of past experiences of discrimination, and a self-reported adherence (Voils) measure.[99, 119] (Table 1) Where patients feel comfortable using a tablet device, surveys will be administered and data will be collected on the tablet via an online survey format and stored in REDCap. If they do not feel comfortable with this format, all surveys will be administered on paper only. The values affirmation exercise will always be administered on paper, and the research assistant will provide instruction and remain available, but the patient will complete the exercise on their own, at their own pace. In our pilot work, patients completed all study materials in ~30 minutes. Next, patients will undergo routine check-in procedures, which includes blood pressure measurement at all sites, and complete the scheduled visit with their provider.

Following the visit with their provider, patients will complete the post-visit surveys that assess the proposed mediator variables, including 1) a standardized measure of patient activation, 2) a measure of attitude, perceived social norms, perceived behavioral control, 3) a measure of anticipated emotion regarding managing their blood pressure, and 4) a visit satisfaction survey.[120, 121] Patients will receive a $20 gift card in compensation for participation. Participants must complete the values affirmation exercise in order to receive compensation for participation. Where patients are not able to complete all other surveys at the baseline/index visit, they will have the option of being contacted by study coordinators via phone to complete any remaining surveys, or to complete surveys via a direct web link to REDCap, that can be sent to them via email.

At 3-months, subjects will be called by the research assistant to schedule 3-month visit which includes measures of blood pressure, pill counts, self-reported adherence survey and the proposed mediators of the intervention effect (Table 1). This visit may coincide with a regularly scheduled follow-up visit with their provider. At 6-months, all subjects will return for an in-person visit to have their blood pressure measured by the research assistant and complete a 6-month survey with the same measures as those obtained at 3-months (Table 1). This visit may also coincide with a regularly scheduled follow-up visit with their provider. At the 3- and 6-month visits, patients will also be asked to bring all antihypertensive medications for pill count assessment, and will receive an additional $20 gift card as compensation for participation. If patients are unable to attend either the 3 or 6-month follow up in person visit, they will be given the option to complete the surveys over the phone, or to complete surveys via a direct web link to REDCap that can be sent to them via email, and their incentive will be mailed to their home.

<u>Provider eligibility</u>. In secondary analysis, we will explore whether provider factors that have been associated with racial attitudes (*implicit racial bias, prior disparities training, self-efficacy regarding* and *interracial mix of clinical site*) influence the association between values-affirmation and adherence.[122] All 200 primary care providers (physicians and advanced practice providers) at the participating 9 clinics will be eligible to participate. Based on our prior work, we anticipate a participation rate of ~60% of eligible providers (n=125).[70] Since the provider measures will be considered separately, patients will be included even if their provider does not complete the survey.

Provider recruitment and procedures. Using methods our team has successfully used in studies surveying healthcare providers, all primary care providers at the participating clinics will receive a formal letter of invitation that includes a brief description of the study, benefits to them, and the endorsements of relevant administrators.[70, 71] The PI, co-investigator Blair and affiliated site co-investigator (Hanratty at DH, Steiner at KPCO, Vupputuri at KPMAS) will then present the project to groups of providers during a regularly scheduled department meeting. Based on our prior work, we know that one hurdle for participation will be busy schedules and therefore we have arranged with clinic directors to give the providers time from the meeting to complete the provider survey measures, online, in the privacy of their offices. Provider participants will receive a $40 gift card code in their invitation letter which they may redeem for their time, and will be provided confidential feedback about their implicit racial attitudes immediately after completing the online survey. The participants will also be given scientific information about how they might minimize the influence of implicit ethnic/racial attitudes on their behavior.[123]

Participants will access the secure website via the link provided in their invitation letter. At the website, they will enter the unique identifier provided to them in their invitation letter, and will then go through informed consent and complete demographic information along with measures of implicit racial bias, assessment of prior disparities training, and self-efficacy regarding caring for African American patients (see Provider Survey). The use of web-based technology will allow the providers to complete the measures privately, on their own schedule, and to retain the confidentiality of their participation. We anticipate providers' concerns about possible sanctions should their biases become known. To overcome these concerns, we will employ methods similar to those used in our prior work that include: 1) developing an elaborate system to ensure providers' complete anonymity, both in terms of their specific responses and with regard to whether or not they have participated; and 2) reassurance that their participation has the full backing of their organization.[20, 70, 71, 113]

C. Study Design and Research Methods

Figure 2: Study Design

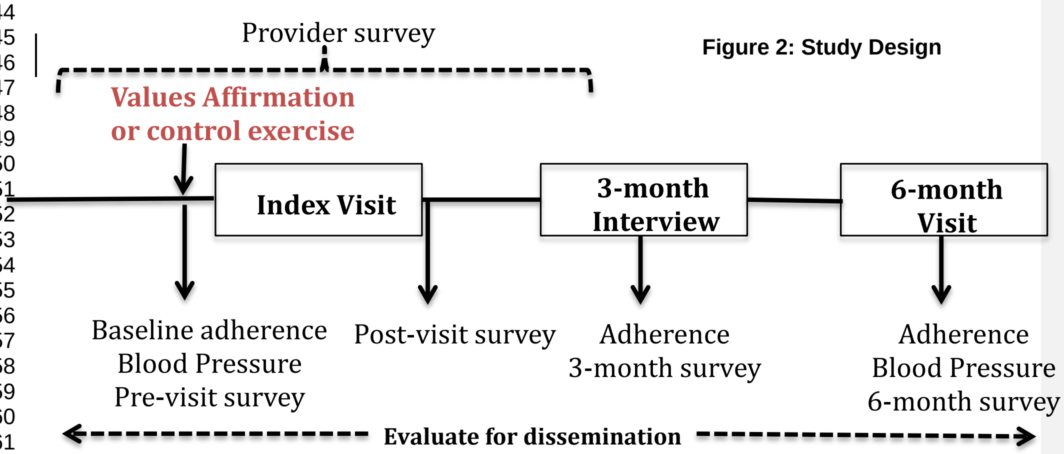

Overview: The study design was developed in consultation with providers at the study sites to ensure feasibility of implementation in routine clinical practice. All primary care providers (PCP) at the participating clinics will be invited to complete a baseline survey regarding past disparities training and racial attitudes; patient participation will be independent of whether their PCP

participates. We will enroll self-identified African American and white patients with uncontrolled hypertension who are eligible and consent for the study. Our primary outcome is medication adherence that will be measured in three ways: 1) pharmacy fills, 2) self-report, and 3) pill counts. At the index visit, before they see their PCP, participants will have their blood pressure recorded and will complete a survey which includes a validated self-report measure of adherence (see Table 1 for survey contents). We will also query the EHR for baseline adherence based on prior 12-months pharmacy fills. Participants will be randomized to the values affirmation or control intervention tasks and they will complete the assigned task before seeing their PCP as scheduled. Following the visit, patients will complete a brief survey. Participants will return at 3-months and 6-months for in-person follow-up visits that will include assessment of adherence, blood pressure measurement and completion of surveys. Participants will be asked to bring all antihypertensive medications to each follow-up visit, and fill date and number of pills in the bottle will be recorded. Adherence will be calculated for each antihypertensive drug in the regimen and combined across drugs into a summary measure of adherence for the entire drug regimen. Secondary outcomes include blood pressure, time under blood pressure control, and appropriate treatment intensification. Throughout the study, we will evaluate the intervention for widespread dissemination based on the RE-AIM framework.[81]

Figure 3: List of Values for the Intervention

1. Sense of humor
2. Religious values
3. Relationships with friends or family
4. Music
5. Politics
6. Membership in a community or social group
7. Living in the moment
8. Independence
9. Creativity
10. Artistic ability
11. Athletic ability.

__Values Affirmation Intervention__. A research assistant will introduce the task to the patient, remain available for questions, but will not otherwise participate in the patient’s completion of the task. The exercise takes approximately 15 minutes to complete. The task first asks patients to reflect on a list of 11 personal values or self-defining skills (Figure 3). Participants are asked to circle the two or three items that are *most important to them* or that characterize them best. The list specifically excludes “good health” or “healthy behavior” because previous research suggests that affirmations in the same domain as the threat can actually increase the threat.[46] Next, participants are asked to think about times when the values chosen were important and then write a few sentences to describe why they were important. They are instructed specifically to focus on thoughts and feelings, and not worry about spelling, grammar, or how well-written the sentences are. Finally, the self-affirmation of values is reinforced by asking subjects to indicate their level of agreement with four statements concerning the selected values using a 6-point Likert scale (strongly disagree, disagree, somewhat disagree, somewhat agree, agree, strongly agree): (1) “These values have influenced my life”, (2) “In general, I try to live up to these values”, (3) “These values are an important part of who I am”, (4) “I care about these values”.

In the ***control task***, participants will receive the same list of values and skills, but will be asked to circle the two or three that are *least important to them*. Controls will receive the same instructions about writing a few sentences about the values chosen, but will be asked to describe when and why these least important values might be *important to someone else*. The final rating task asks subjects to indicate their level of agreement using the same 6-point scale with slightly altered statements: (1) These values have influenced some people, (2) Some people may try to live up to these values, (3) These values may be important to some people, (4) Some people care about these values. After the task, subjects will place their responses in an envelope, seal them, and return them to the research assistant. It will be made clear to subjects that only study personnel and not their providers will see the responses. The exercise will not be repeated on subsequent visits.

Patient measures. Using validated instruments, we will assess for patients' prior experience of discrimination that might moderate the interventions' effects. We will also assess for cognitive factors that might mediate the interventions' effect on adherence: patient activation and constructs derived from a modification for hypertension of the Theory of Planned Behavior: attitude, perceived social norms, perceived behavioral control, and anticipated emotion. (Table 1) Finally, we will assess patient's impression of the visit.

Table 1: Patient survey contents

Pre-visit Survey	Post-visit survey	3- and 6-month surveys
Prior discrimination	Patient Activation Measure	Patient Activation Measure
Voils adherence questionnaire	Attitudes Survey	Attitudes Survey
	Patient impression of the visit	Voils adherence questionnaire

Prior experience of discrimination. We will treat perceived discrimination as a potential modifier of the effect of the intervention on adherence. We will use a 4-item questionnaire about prior experience of discrimination modified from prior scales measuring lifetime discrimination related to healthcare.[124-128] Responses are on a 5-point Likert scale ranging from "never" to "all of the time". (see Prior Discrimination Survey- new version)

Patient activation. We will also assess the effect of the intervention on patient activation, our theoretical mediator of the effect of values-affirmation on engaging in hypertension care.[64, 65] (Figure 1) We will use a low-literacy version of the 13-item instrument developed by Hibbard, the Patient Activation Measure (PAM).[120] Increases in the PAM are associated with increase self-management behavior including adherence.[64, 65, 129] Patient activation measured with the PAM is lower for African Americans than it is for whites, and is therefore hypothesized to be a contributing factor to racial health disparities.[130] Few interventions have been shown to be effective for increasing patient activation. Each questions is answered on a 4-point Likert scale.

We will treat the PAM score as a potential mediator of the effect of the interventions on adherence and measure this construct after the index visit and at 3- and 6-months to determine maintenance of effect.

Attitudes Survey. In order to explore other potential mediators of the relationships between values affirmation, improved patient-provider communication and improved patient intention for self-care, we rely on constructs from the Theory of Planned Behavior as modified by Perugini and Bagozzi and tested in patients with hypertension.[121, 131] The Theory of Planned Behavior posits behavior intention as the most proximal predictor of behavior, and that intention is influenced by the individual's attitude towards the behavior, by the social norms surrounding the behavior, and by the individual's perceived control over the behavior.[132] Taylor and Bagozzi modified this theory by adding the concept of desire for the behavior and its consequences, which in turn is influenced by the anticipated positive and negative emotions associated with the behavior.[121] We have adopted the questions from this study to assess attitudes, norms, perceived control, and emotions around blood pressure management. (see Attitudes Survey) We will add an additional question to include physicians' opinions in the patients' perception of social norms: "I care what my doctor thinks about my efforts to control my blood pressure", with responses ranging from "not at all" to "very much" along a 5-point scale.

Patient assessment of visit – As in our pilot study, as a secondary outcome, we will use the Barr's 4-item provider specific modification of the Medical Outcomes

Study Visit Satisfaction Questionnaire, which is sensitive to differences in patient satisfaction by race.[133]

Provider Measures. – We will assess provider factors that may moderate the relationship of the intervention with adherence including implicit racial bias, prior cultural awareness training, self-efficacy regarding caring for African American patients and interracial mix of clinical site. These measures will be included in a web-based survey and were chosen based on literature suggesting these factors influence the quality of the patient-provider interaction and were associated with differences in racial attitudes among health care providers.[60, 61, 122] This survey will be completed by providers at their convenience and independent of the patient index visit. (see Provider Survey)

Implicit racial bias – We will use the Black-White Implicit Association Test (IAT) to measure providers' racial bias. The Black-White IAT uses reaction times to assess the strength of automatic associations between race (black, white) and evaluations (e.g. positive words, negative words). Implicit bias against blacks compared with whites is shown if one is significantly faster when black faces and negative words require the same response while white faces and positive words require another response, compared with the reverse pairing. The larger this performance difference, the stronger the implicit bias (see https://implicit.harvard.edu). The IAT has been widely validated, is reliable over time and is associated with discriminatory judgments and behaviors.[123, 134-140] Our research group has successfully used the IAT among health providers and we have demonstrated an association between IAT scores and patient assessment of their provider and some health outcomes.[20, 70, 71, 113]

Past cultural competency training – We will ask participating PCPs whether they have ever participated in a course, seminar, or workshop on minority health. We will ask a similar question regarding past exposure to cultural awareness or diversity training.[122] For those who respond positively to either question, we will further probe on the frequency and timing (e.g. within past 0-1, 1-5, >5 years) of this training.

Self-efficacy regarding providing care to African Americans – Based on work by van Ryn et al. demonstrating self-efficacy regarding providing care to African American patients was associated with changes in racial bias among medical students, we will ask participating providers to report on the degree to which they feel: 1) 'prepared to handle a patient who is a member of a racial or ethnic minority'; 2) 'skilled at overcoming unintended or unconscious racial bias'; and 3) 'skilled in developing a positive relationship with racial minority patients'.[122] Each question will be answered on a 4-point Likert scale.

Interracial contact at clinical site – Prior studies have suggested that the amount of interracial contact influences interracial attitudes and behaviors.[122, 141-143] At each clinical site, administrative data will be used to determine the racial mix (proportion of African American versus white) patients and clinical staff (physicians, advanced practice providers, and nurses).

Evaluate for dissemination, RE-AIM. The RE-AIM (Reach, Effectiveness, Adoption, Implementation, and Maintenance) framework will be used to evaluate the values affirmation invention in order to enhance the quality, speed, and public health impact of translating the intervention into widespread clinical practice (**www.re-aim.org**). Dr. Glasgow, an ACCORDS investigator (primary research site) developed the RE-AIM model as an evaluation framework to expand assessment of interventions beyond efficacy to multiple criteria that may better identify the translatability and public health impact of interventions and that balances the emphasis on internal and external validity.[82, 83] We will apply RE-AIM by the examining the following:

Reach - the participation rate and representativeness of individuals who participate in the project. The participation rate is the proportion of patients who are approached who agree to participate in the study. The demographic and clinical factors of those who participate and those who do not will be compared to the same factors of the larger

population of patients with hypertension. Finally, we are deploying the intervention within three large health systems with varied demographic characteristics to increase the external validity.

Effectiveness – the impact of an intervention on outcomes**.** Since adherence is the target of the intervention, it will be the primary measure of effectiveness. Adherence will be measured at baseline and at 3- and 6-months to assess the impact and sustainability of the intervention at improving adherence. We will compare the effectiveness of the intervention to a control exercise in both African American and white patients to determine if the effect is dependent upon race. As discussed in the patient and providers' measures sections, we will also examine potential moderators of the intervention to understand whether the intervention is similarly effective across all groups. We will also compare the effectiveness according to baseline adherence and baseline blood pressure control.

***Adoption* –** the proportion and representativeness of the intervention setting (e.g. clinics, physicians) who are willing to accept the intervention. Our initial inquiries indicate that all DH and KP clinics that are approached will be interested and willing to participate in the program and thus we expect adoption at the clinic and physician level to be 100%. However, we will confirm whether this occurs and to the degree that it does not, we will compare the characteristics of the clinics and physicians who do participate to those who do not.

Implementation - the extent to which the intervention is delivered as intended. We will ask patients, providers and study personnel about barriers and facilitators to implementation. We will also evaluate the written comments by patients to determine if the patients performed the writing exercise per instructions. Since time is often one of the key factors in determining how widely an intervention will be implemented in practice, we will formally track the time it takes to complete the intervention to the end of the visit and examine whether the intervention interferes or delays patient flow through the clinic.

Maintenance – To assess the maintenance or decay of the intervention effect, we will assess the primary measure of effectiveness (adherence) at both 3 and 6 months after the single intervention. Using the pharmacy records, we also have the ability to evaluate adherence beyond 6-months.

Timeline. We will stagger the initiation of enrollment across the three sites for two reasons. First, study procedure difficulties are inevitable. Identifying them and working out solutions is far easier at one site than three. Since the pilot study took place at DH, initiating enrollment there will require less education and start-up time. The 30-month recruitment period at each of the three sites is conservative (14 patients per month per site, or 4-5 patients per clinic per month for a total estimated sample size of 1130), but is based on our experience in the pilot study, in which the subject accrual rate was approximately 12 patients per month at a single site. The 6-month follow-up period reflects adherence determination for the last patient enrolled. (Table 2)

**Table 2**	Year 1				Year 2				Year 3				Year 4			
IRB																
DH		Recruitment										F/U				
KPCO			Recruitment										F/U			
KPMAS				Recruitment										F/U		
Analysis																

D. Description, Risks and Justification of Procedures and Data Collection Tools:

The primary research site is the Adult and Child Center for Health Outcomes Research and Delivery Science (ACCORDS) at the University of Colorado School of Medicine. Patients will be recruited from three health systems: Denver Health (DH), Kaiser Permanente Colorado (KCPO) and Kaiser Permanente Mid-Atlantic States (KPMAS). We will submit applications to three Institutional Review Boards for approval to conduct this research: (1) the Colorado Multiple Institutional Review Board (COMIRB), which serves as the IRB for the University of Colorado,

ACCORDS and Denver Health; (2) the KPCO IRB; and (3) the KPMAS IRB. We will coordinate these submissions (and any necessary changes) until we have obtained agreement and approval from all boards.

1. Risks to human subjects

a. Human Subjects Involvement, Characteristics, and Design: As described in the Research Strategy, the intervention to be evaluated under this application is solely directed at human subjects. We will conduct a randomized controlled trial at the patient level. Providers will be separately recruited for a survey study. Patient participation is independent of provider participation.

Patients - As described in the Research Strategy section we will enroll 1,130 ambulatory patients over the age of 21 with uncontrolled hypertension that is not associated with pregnancy or with end-stage renal disease who receive regular care at nine outpatient clinics. Additional inclusion criteria are ability to speak and write English and self-described race of African American or white, and a plan to obtain antihypertensive medication from the health system. Sampling is done by convenience, with potential subjects identified from lists of patients scheduled for clinical visits in the upcoming week and eligibility assessed by chart review. No special classes of vulnerable subjects will be enrolled. Assignment to study group will be done at random. Management and protection of data is described in section 2b below.

Providers - All 200 primary care providers (physicians and advanced practice providers) at the participating clinics will be eligible to participate in the survey study. Given estimates of the participating health systems, we anticipate that approximately 60% of primary care providers will be female and 80% will be self-identified as white. These participants are anticipated to be in the age range of 27 – 80 years old. No subpopulation of these providers will be excluded as long as they can complete the study tasks, which will be in English. Providers' health status is unknown. No specific inclusion or exclusion criteria will be placed based on health status.

b. Sources of materials: Data will be collected for research purposes only. The data coordination and analysis will be based at the University of Colorado ACCORDS program. ACCORDS has the staff (including Drs. Daugherty, Havranek and Dr. Dickinson), equipment, and experience necessary for the coordination and analysis of study data.

Patients - Data to be obtained from patients are their statements of values obtained as part of the self-affirmation intervention, patient age, gender, and race as reported by the patients, medications obtained from administrative data, responses to the visit questionnaires, adherence data obtained from administrative data or electronic monitoring, and blood pressure measured at baseline and follow-up visits. All material obtained directly as part of the study will be entered into a computerized database in which the only identifier will be a unique study identification number; linkage of this unique study identification number to the patient's name and medical record number will be kept in a separate database. Data from the former database will only be accessible to study personnel.

Providers - Data sources will be a web-based survey of providers' racial attitudes (Black-White Implicit Association Test), self-report of past cultural competency training, and self-report of efficacy caring for minority patients. Providers will be given a unique study identifier to use as a password in accessing the web-based survey. The site data analyst will retain the list of names linked to the identifiers, which will not be shared with the other research support staff or investigators. Only these identifiers will be used to track participation and link patients to their providers for analytic purposes.

c. Potential risks: Every possible effort will be made to minimize risks and discomforts to participants. Nevertheless, remaining risk is expected to consist of-

Patients - The intervention itself does not pose known risks to subjects. There is potential risk to patients in that they may feel pressure to participate, and in that confidential personal/medical information is being collected and stored. There is also the risk of evoking an emotional or negative response to the writing exercise.

Providers - The primary risk for providers is inadvertent or unauthorized release of information about their implicit racial attitudes. A secondary risk is the potential for the measures of implicit gender bias to create psychological distress, upon learning that one's implicit biases might be stronger than one would like.

2. Adequacy of protection against risks

a. Recruitment and informed consent:

Patients - Eligible patients will be approached prior to their scheduled clinic visits. Since investigators will have established relationships with the potential patients, screening for eligibility will occur prior to the clinic session. Our recruitment/retention strategy includes a $20 gift card for the initial visit and $20 at the 3 month and 6 month follow-up visits. We believe this form and amount of compensation to be high enough to be fair and low enough not to be coercive.

Informed consent will be obtained by the research assistants in a clinic examination room in order to allow the process to be private and confidential. Following elucidation of the nature, risks, and possible benefits of the study, subjects will be asked to sign written informed consent as approved by the Institutional Review Board for the health system the subject is engaged with (DH, KPCO, KPMAS). Additional options will be provided to request consent to contact participants for future studies, and consent to contact an additional contact phone number (provided by participants) if the baseline contact phone number becomes unavailable at the 3- or 6-month follow-up time point. Participants may choose not to provide additional contact numbers and can opt-out if not interested/they do not agree to participate in future studies. We are cognizant of the potential for eligible research subjects to feel pressured to participate (risk of coercion) given that some will be medically indigent and therefore have few options for obtaining primary medical care outside the clinic. We will address this potential in two ways. First, the process of informed consent will make explicit that the decision on whether or not to participate will in no way affect current or future care. Second, the consent process will be carried out by research staff, not clinic personnel, further de-coupling the research and their usual care.

Providers - Using methods our team has successfully used in studies surveying physicians, all primary care providers at the participating clinics will receive a formal letter of invitation that includes a description of the study, benefits to them, and the endorsements of relevant administrators. The PI, co-investigator Blair and affiliated site co-investigator (Hanratty at DH, Steiner at KPCO, Vupputuri at KPMAS) will then present the project to groups of providers during a regularly scheduled department meeting. Based on our prior work, we know that one hurdle for participation will be busy schedules and therefore we have arranged with clinic directors time after the meeting to complete the provider survey measures, online, in the privacy of their office. All invited providers will receive a $40 gift card incentive regardless if their decision to participate. Participants will also receive immediate confidential feedback about their implicit racial attitudes. The participants will also be given information about how they might minimize the influence of implicit racial attitudes on their behavior.

Informed consent will be administered at the website. After using their unique study identifier to log-on to the study website, the providers will receive a series of "pages" of informed consent and need to click on an "I consent" button to continue. Failure to click

the consent button will result in the termination of the study (participants have the option of returning to the site and, with completed informed consent, participate in the study). Providers will be informed about the goals and procedures of the research including information about both their direct participation (i.e., completing the online survey) and the participation of their patients (i.e., associating their attitude data to patients' data). No signature or any other paperwork will be collected from the provider participants, thereby eliminating identification from sources outside of study personnel. Similar methods of consent have been approved by COMIRB for Dr. Daugherty and Dr. Blair's prior studies measuring implicit bias among physicians. We estimate providers will take approximately 15 minutes to complete the study measures. The data provided by the providers will be used for both descriptive purposes and in the moderation analysis.

b. Protection against risks:

Patient - Every possible effort will be made to protect subjects from risks and discomfort. At the time of consent, all participants will be given contact information for study personnel and community mental health resources. Further, the patient will see their primary care provider immediately after the exercise and the provider will be notified of any negative responses to the intervention.

The risk of inadvertent or unauthorized release of confidential participant information will be protected against in a number of ways. As general steps, all paper documents will be stored in a locked room inaccessible from general traffic areas whose sole purpose is storage of clinical research material. All electronic documents will be stored only on ACCORDS, DH, KPCO, or KPMAS computers, which are subject to strict security requirements standard for healthcare institutions. Data will be analyzed under the supervision of Dr. Dickinson by the analytic group at ACCORDS according to policies developed to address the needs of prior NIH-approved studies in which patient data gathered at other health systems are analyzed at ACCORDS. In brief, data transferred to ACCORDS will have only one patient-specific identifier, a study identification number, and will not contain patient name, medical record number, social security number, address, or date of birth (age in years only). The file linking the study identification number and other patient identifiers will exist only at DH, KPCO, or KPMAS. This methodology de-identifies most, but not all patient files. For instance, there may be fewer than ten patients with hypertension of a given age at the study site. Therefore, we have taken measures to ensure the confidentiality of data transferred to ACCORDS. Data are transferred only via an encrypted e-mail link. Policies at all participating institutions prohibit data storage on portable media including laptops, external hard drives, CDs, or flash drives. Once transferred to ACCORDS, data are stored only on a server, never on desktops or laptops. Data files are password protected. Only the ACCORDS director grants access to data files. The data security standards for the ACCORDS server are administratively the same as that for the University of Colorado Hospital's system for patient data. Data will be deleted from the DH, KPCO, KPMAS, and ACCORDS servers five years following completion of the analysis of the data.

Providers - As noted above, there is the potential for some psychological distress on the part of providers who learn that their implicit racial biases might be stronger than they would like. Dr. Blair (co-investigator for this award) has over 15 years of experience in conducting research on implicit biases and has not had a single adverse reaction. Although research subjects are sometimes surprised by their implicit biases, they have generally reported that the educational experience outweighed any distress they experienced. We will take steps to minimize any psychological distress. Specifically, research subjects will be informed about the prevalent nature of racial biases and the scientific understanding that such biases are often the unintended result of normal social processes, such as a healthy social identity and the culture-specific socialization people receive. Subjects will also be informed that biased attitudes need not necessarily translate into biased behavior,

and they will be given information about how they can ameliorate the effects of bias on their behavior.

The web-based survey will be developed with the assistance of the University of Colorado Office of Information Technology to ensure appropriate security measures and participant confidentiality. The University of Colorado has a detailed system of data protection and numerous layers of data security. The web server will use encryption for data transfer between the respondent's computer and the research server. All research data will be stored on an ACCORDS server and can only be accessed by research personnel using their personal secure login and password. As a further protection of participant confidentiality, all potentially identifying information (IP address, email address) will be housed in data tables that exist separately from research data collected at the website. This will allow research staff to track participation and avoid duplicate data, but will prevent direct access to view that identifying data. Each patient participant file sent to ACCORDS from the clinical sites will include their provider identified by the provider’s study ID. The ACCORDS analyst will merge the raw data from the provider survey to the patients linked to that provider. Once the merge is complete, these identifiers will be replaced with randomly generated codes, leaving no record that connects the new codes to the former study identifiers. The records will be further deidentified by substituting randomly-generated codes and collapsing any demographic data for subgroups that could be used to identify fewer than 5 individuals. Thus, in the event that there is inadvertant or unauthorized release of data, there will be no way for any person or the health systems to identify individual providers’ data. In the absence of any written record of participation (i.e., no signed informed consent forms), there will be no way for anyone within the health systems to even know which providers participated in the study.

3. **Data and safety monitoring plan**: Conduct of and oversight for the study according to NIH policy are the responsibility of the investigators. The investigators are directly accountable for this conduct and oversight to the Colorado Multiple Institution Review Board (COMIRB) which oversees research at the University of Colorado School of Medicine and DH, and to the separate institutional review boards for KPCO and KPMAS. As previously noted, all three institutional review boards must approve the study in order for it to move forward.

DSMB

In addition to oversight by the principal investigator and the institutional review boards, we have put in place a Data Safety Monitoring Board (DSMB). The board has three members: 1) Dr. Raymond Estacio, Professor of Medicine, primary care physician and expert in clinical trials from the Colorado Prevention Center Clinical Research unit; 2) Dr. William Henderson, Professor and biostatistician from the Colorado School of Public Health Department of Biostatistics and Informatics; and 3) Dr. Frederick Masoudi, Professor of Medicine and cardiology physician with expertise in health services research from the University of Colorado. The proposed membership ensures the relevant expertise is present and that none of the DSMB members are members of the investigative team. The DSMB will review the protocol prior to implementation. Subsequent meetings will be held in person or via teleconference at least once a year with additional meetings or conference calls scheduled as needed. The study manager in collaboration with both PIs and NHLBI Program Officer will schedule meetings and conference calls. We will ask the board to review evidence of study-related adverse events, data quality and completeness, and adherence to the protocol. As stated above, because there are no known adverse effects associated with the planned intervention, we plan no interim analysis of the data. We must reserve the right, however, to consider interim analyses should the DSMB request this be done.

Data collection and management

Data warehouses (DW) - DH and the KP health systems maintain extensive patient data in a series of standardized data warehouses that are available for research and evaluation

applications.[144] DH's data warehouse includes integrated administrative and clinical data as well as disease management registries for conditions such as hypertension and diabetes. Within KP, the Virtual Data Warehouse (VDW) standardizes clinical, utilization and demographic research data into a common structure shared by all members of the Kaiser Permanente health system.[144] The VDW facilitates collaborative efforts with other KP sites by enabling a single analytic script to be used to extract and process data at each research site, significantly reducing the effort and time required to conduct multi-site studies. The data collected and maintained in both systems includes: membership and demographic information, clinical office visit procedures (CPT-4 codes) and diagnoses (ICD-9 codes), pharmacy dispensing, radiology procedures, lab tests and results, patient office visit registration, hospital inpatient and outpatient data, and health care provider information. Historical data in both systems go back to 2004, with some data available as far back as 1995.

Our research team has successfully standardized data definitions from DH and KP data warehouses on prior hypertension studies.[20] As part of this prior work, data elements and data dictionaries relevant to hypertension (i.e. blood pressures, antihypertensive medication names, classes, doses) have been created which specify a common format for each of the elements—variable name, variable label, extended definition, code values, and value labels. Local site programmers have mapped the data elements from their health systems data onto this standardized set of variable definitions, names, and codes, as well as onto standardized SAS file formats. This common structure of the files enables a SAS analyst at one site to write one program to extract and/or analyze data at all participating sites. The program will be emailed to programmers at the sites to run against their own data warehouse files and the resultant de-identified data are transferred to the analytic site via a secure encrypted Web site.

Data collection

This project will produce a variety of data types across the four years of the project. These include: (a) survey data, (b) blood pressure measurements, (c) pill count data, and (d) information pulled for the study sites DW (e.g. BPs, diagnoses, pharmacy fills). To ensure data integrity, the study protocol manual will include a section on data collection with descriptions of each data element or measure and instructions for its accurate collection or acceptable source. A list of proposed study variables, how they will be used, when they will be collected and their source are included in the table.

Data management - Study data will be obtained from the sources noted in Table 3. A unique study ID will be assigned to each participant. The electronic list that matches names or clinic IDs to study IDs will be password-protected and available only to the project team at each site. A tracking database on a secure server will monitor data collection and storage. The study coordinator at each site will enter additional information (e.g., date of baseline visit) and produce periodic reports that identify participants due for their follow up visit. Data quality will be assessed using standard and project-specific quality checks at the recruitment sites and the data-coordinating center.[144]

Data will be sent from the study sites to the data-coordinating center at ACCORDS using a secure file transfer site maintained by UCD. This site secures files received from, or sent to, sources outside UCD. The recipient, internal or external, receives a notification when files are available to download and the system maintains an audit trail of all transactions. Data sent to ACCORDS will be transferred in a timely manner to internal servers that are maintained behind a second firewall with additional restricted access. This procedure has been used successfully in many other studies.

Table 3. Proposed Study Variables to be collected at each site

Variable	Use	Timing	Source
Refill adherence	Primary outcome	Ongoing	DW - pharmacy records, using summary measure of proportion of days covered[90]
Pill counts for adherence	Primary outcome	Baseline, 3- and 6-months	Measured at study visits by trained personnel
Self-reported adherence	Primary outcome	Baseline, 3-and 6-months	Patient completed survey
Systolic and diastolic BP	Secondary outcome	Baseline and 6 months	Measured at study visits by trained personnel
Time under BP control	Secondary outcome	Ongoing	DW - Measured in routine care
Appropriate Treatment intensification[106]	Secondary outcome	Ongoing	DW – Medication class additions or dose increases
Prior experience of discrimination [124-128]	Potential Moderator	Baseline	Patient completed survey
Provider implicit racial bias[140]	Potential Moderator	Baseline	Provider completed survey
Provider past disparities training.[122]	Potential Moderator	Baseline	Provider completed survey
Provider self-efficacy regarding care to African American patients.[122]	Potential Moderator	Baseline	Provider completed survey
Provider interracial contact.[122]	Potential Moderator	Baseline	Health system administrative data
Patient activation measure (PAM)[120]	Hypothesized Mediator	Baseline, 3- and 6-months	Patient completed survey
Attitudes survey[121, 131]	Alternative Mediator	Baseline, 3- and 6-months	Patient completed survey
HTN medications	Covariates	Ongoing	DW - pharmacy records and medication lists
Relevant comorbid conditions (diabetes, atherosclerotic cardiovascular disease, heart failure, stroke, kidney disease)	Covariates	Ongoing	DW, Problem list, claims, clinical diagnoses from visits
Relevant risk factors (Tobacco, alcohol and controlled substance use)	Covariates	Ongoing	DW - Problem list, claims, clinical diagnoses from visits
Socio-demographic factors (Age, gender, education, income, employment)	Covariates	Baseline	DW- Enrollment files
Insurance payer(s)	Covariates	Baseline	DW - Enrollment files
Contact information (address, landline and cell numbers, contact person)	Eligibility criteria, recruitment	Baseline	DW - Enrollment files

*DW = Data warehouse at each clinical site; MEMS = Medication Event Monitoring System

4. **ClinicalTrials.gov requirements**: Although not required by law, we will register our study.

E. Potential Scientific Problems:

As in any clinical trial, the potential problem that supersedes all others is inadequate patient enrollment. Our best alternative strategy for overcoming this is to use additional clinics; each of the 3 health systems has multiple clinics in addition to the sites we are targeting. If enrollment is low at a chosen site, we will expand to another clinic within that system.

At one site, a similar COMIRB-approved study will also be recruiting white patients with hypertension thus reducing potential enrollment in our study. The patients enrolled in this second study complete similar survey instruments, receive the same intervention or control exercise, and have similar data collected (blood pressure readings and measures of medication adherence) as what is proposed in our current study. These data are collected on a timeline identical to our study schedule (baseline, 3 months, and 6 months).

Rather than "competing" with this second study for patient enrollment at this site, where a relatively small percentage of patients are white, we propose a dual enrollment plan. Under this plan, both studies would share data collected among enrolled white patients at this site. Patients would thus sign two informed consent forms, but only complete surveys and provide other collected data one time. Therefore, there would be no additional burden to participants. We believe this strategy will significantly improve our accrual of subjects and will not compromise the scientific merit or integrity of either study.

We acknowledge alternative patient-centered approaches may further mitigate the negative effects of stereotype threat such as increasing the numbers of minority clinicians, posting diversity mission statements and training providers to provide motivating feedback.[21] However, all require significant resources to implement and are beyond the scope of this research study. We propose that a simple, low cost intervention allowing patient's to affirm his or her valued characteristics, will have important, immediate effects.[21]

Another potential problem is the racial mix of providers. While at DH, there are no African American physicians; both the KPCO and KPMAS sites have African American providers (10% KPCO and 20% KPMAS). Values-affirmation is expected to have a lesser effect or no effect at all when both patient and provider are African American, because the threat of being judged according to negative stereotypes is lessened when the person in power is a member of the same group. We will not, however, exclude patients of African American providers, and recognize this will slightly diminish our ability to detect the difference hypothesized under Aim 1. We have made this decision because we think it is important to be able to report how the intervention performs when the provider group is mixed to increase external validity. We will explore the effects of provider race in secondary analysis.

Finally, we recognize that the proposed intervention does not target bias and prejudice on the part of providers. Instead, values affirmation works by empowering the patient to overcome the negative effects of perceived discrimination in the health care setting. As we have discussed above, at this time, there are no known effective interventions for provider bias and this is an area of future research interest for our group. We will measure provider racial bias and past cultural competency training to examine how these factors potentially modify the relationship between values affirmation and patient outcomes.

F. Data Analysis Plan:

Sample size estimation. For our primary analysis, we assume a three-level model, with patients nested within providers and providers nested within clinics. Assuming comparable variation in adherence and α=0.05, a sample size of 960 subjects (240 each cell in values affirmation, or control; 480 African American, 480 white) will be required to detect an effect size of 0.26 difference in adherence between any two cells with a power of 80%. Based on the pilot study results, a 0.26 effect size is approximately a 4.7% absolute difference in adherence scores. Since randomization is at the patient level, the intraclass correlation coefficient for patients nested within clinicians will

have a negligible effect on power. We conservatively estimate an attrition rate (neither having a med refill within the system nor returning for follow up) over all sites of 15%. We therefore will enroll an additional 170 patients to compensate for this attrition, for a final sample size of 1,130. We will cap enrollment at 450 subjects per site to ensure generalizability of results.

For moderation hypotheses, the primary contrast of interest is the adherence difference between African Americans with high and low past experiences of discrimination (dichotomized scores for ease of interpretation) who receive the intervention. Using a median split on the discrimination scores, the sample size for this comparison would be 120 in each subgroup. This sample size will provide >80% power to detect a 0.37 SD difference in adherence. For the patient activation mediation analyses, a sample size of 480 (e.g. African Americans only) will provide >80% power to detect a 0.13 partial correlation between change in the patient activation and change in adherence, adjusting for other variables in the model; a sample size of 960 will provide >80% power to detect a .10 partial correlation. Parchman et al report a standardized regression coefficient of 0.16 between patient activation and the Morisky medication adherence scale.[64, 145] While our proposed analytic approaches differ, this gives some indication of the anticipated strength of the relationship.

For the secondary outcome measure of blood pressure, this sample size has a power of 80% to detect a 4.7 mmHg difference in systolic blood pressure between any two cells. For perspective, in a study of a pharmacist-led multimodal intervention, an increase in adherence from 62% to 97% (a 35% absolute increase) was associated with a decline in systolic blood pressure from 133.2 to 129.9 mmHg (a 3.3 mmHg absolute decrease).[16] Thus, the study is likely underpowered to detect a difference in systolic blood pressure through adherence change alone. Given the established link between higher adherence rate and improved clinical outcomes, we believe a study powered to detect an adherence difference will be sufficient evidence to move this intervention forward into clinical practice.[88, 89, 146]

Statistical Analysis. Descriptive statistics (Chi-square and t-tests) will be computed to determine whether there are differences between patients randomized and not randomized, between patients randomized to different study arms, or between dropouts and non-dropouts.

The primary outcome variable (adherence measured by pharmacy refills) is continuous; in the event that normality assumptions are not met we will use transformations to normalize distributions. For the primary comparisons, we will employ intent-to-treat analyses, although we expect few or none of the randomized patients to not complete an exercise. We will examine overall values affirmation effectiveness between the racial groups. We will next create models with adherence as the dependent variable including the potential moderator variable (prior experience of discrimination) then including the potential mediator variables (patient activation, attitude, social norms, perceived behavioral control, and anticipated emotion).[147] Finally, we will explore whether the collected provider variables (racial bias, past cultural competency training, self-efficacy and racial mix of clinical site) moderate the relationships. We will also include patient gender and provider race as a possible moderating variables. We will use general (or generalized, for dichotomous outcomes) linear mixed models, examining alternative structures for the variance-covariance matrix for the level 1 model by comparing goodness of fit for the different models as well as model fitting diagnostics to assess for influential points, outliers, overdispersion and heteroscedasticity. We will employ methods that utilize all available data, assuming ignorable missingness.

In secondary analysis using the self-reported adherence measures, we will dichotomize the groups into non-adherent (score ≥ 2 on any “extent” item) and adherent. We will also compare the pill count measure of adherence to the pharmacy fill estimates of adherence for validation. For blood pressure, the secondary outcome, we will compare the index and follow-up visits using mixed effects repeated measures models with a patient random effect. All hypothesis tests will be two-sided with alpha=.05. Statistical analysis will use SAS 9.4.

***Statistical Model for Primary Outcomes*.** The structure of the data is hierarchical (patients nested within providers) and longitudinal (repeated measures on patients over time). Baseline (time 0) is defined as the adherence rate for the 12-months prior to the index visit.

Level 1 model. Repeated measures within each patient will be modeled as a growth curve model (shown here) or repeated measures. Time will be coded 0 for baseline and days since baseline for all post-baseline measures. The outcome for patient i measured at time t in practice j is Y_{tij}

$$Y_{tij} = \pi_{0ij} + \pi_{1ij}\,(\text{time})_{tij} + \varepsilon_{tij}$$

where π_{0ij} is the individual status at time 0, π_{1ij} is the linear growth rate for patient ij, and ε_{tij} is the term that represents the random deviation of observation t within patient ij from the predicted value.

Level 2 model. The patient level models specify the relationship between the patient-level coefficients and the coefficients in the Level 1 model. Intervention status will be coded 1, if intervention, 0, if control. Race (AA) will be coded 1, if African American; 0, if white. Interaction terms will be included to estimate differential intervention effects for AA vs white. Fixed patient-level covariates (not shown) will be included at this level. $\pi_{0i} = \beta_{00} + \beta_{01}$ (Interv) + β_{02} (AA) + β_{03} (Interv x AA) + r_{0i}

$$\pi_{1i} = \beta_{10} + \beta_{11}\,(\text{Interv}) + \beta_{12}\,(\text{AA}) + \beta_{13}\,(\text{Interv x AA}) + r_{1j}$$

where β_{00j} represents the initial status of white, control patients within clinician j, $\beta_{01,}$ β_{02}, and β_{03} represent baseline differences by intervention status and race. β_{10j} represents the linear growth rate for white control patients within clinician j, β_{11} represents the difference in slope for white intervention vs controls, β_{12} represents the difference in slope for AA vs white control patients, and β_{13} represents the differential intervention effect for AA vs white patients within clinician j. The r's are patient-level random effects.

Level 3 model. The provider level models specify the relationship between the provider-level predictors and the coefficients in the Level 2 model.

$$\beta_{00j} = \gamma_{000} + u_{00j}, \quad \beta_{01} = \gamma_{010}, \quad \beta_{02} = \gamma_{020}, \quad \beta_{03} = \gamma_{030}$$

$$\beta_{10j} = \gamma_{100}, \quad \beta_{11j} = \gamma_{110}, \quad \beta_{12j} = \gamma_{120}, \quad \beta_{13j} = \gamma_{130},$$

where γ_{000} is the intercept in the provider level model for β_{00j} (i.e. initial status for white controls); γ_{010}, $\gamma_{020,}$ and γ_{030} represent overall baseline differences by intervention status and race. γ_{100} is the linear growth rate for white controls; γ_{110}, $\gamma_{120,}$ and γ_{130} represent differences in slope by intervention status, race, and both. u_{00j} is a provider random effect. Thus, hypotheses can be tested directly by examining coefficients for the terms above or by means of linear contrasts.

Mediator analyses for the adherence outcome will use methods that completely partition between and within patient effects, with *change in adherence* as the primary outcome and *change in PAM scores* (or other measure) as the primary independent variable.[148]

In addition to the planned analyses above, several additional analyses will be conducted using the study's currently collected data. No new data or variables will be collected. The results of the analyses will be included in abstracts for meetings/conferences, and/or in manuscripts submitted for publication. The list of additional analyses are below:

1. Social vulnerabilities to disparities and participant attrition: To explore whether social vulnerabilities are associated with participant attrition in a randomized trial, we will examine the relationship between age, minority race-ethnicity, sex, financial strain, insurance status, unemployment and educational attainment with enrollee attrition over 6 months.
2. Racial differences in theory-based constructs related to hypertension management: To understand the extent to which attitudinal constructs predict adherence behavior across racial groups, we will estimate a model in which prior adherence is the primary variable that predicts current attitudes towards adherence, norms supporting adherence, and perceived

behavioral control over adherence. Perceived discrimination will be explored as an additional predictive measure. Future analyses may be similar to those listed above but will instead examine the value of attitudes, norms, and perceived behavioral control in predicting medication adherence for each of the racial groups at 3- and 6-month follow-up.

3. Racial differences in values and qualitative themes: We will use quantitative Poisson regression models and qualitative content analysis to determine the frequency of values chosen during the values affirmation writing exercise and identify common qualitative themes for why each value was chosen among racial groups.
4. Past experiences of discrimination and uncontrolled blood pressure: We will determine whether there is an association between past experiences of discrimination and the degree of uncontrolled blood pressure at patients' index visit. If so, we will examine potential moderators of that relationship (satisfaction with clinician, patient activation, attitudes toward blood pressure control) while controlling for socioeconomic status and treatment variables.
5. Pill management strategies and pill count adherence: To investigate how strategies used by patients to manage their medications outside the clinic are associated with adherence to medications, we will describe the self-reported frequency of strategies used by patients to manage their pills at home. The association between each strategy and patients' pill count adherence (proportion of actual over expected pills taken) will be determined at each study timepoint. In secondary analyses, the strategies associated with apparent outliers will be examined at the baseline visit.
6. Factors contributing to the racial disparity in antihypertensive medication adherence: To assess whether racial disparities in medication adherence can be explained by pre-specified covariates, we will use the Oaxaca-Blinder decomposition method. We will determine general sample characteristics of the patient population who are adherent and non-adherent to antihypertensive medications, determine the overall disparity in medication adherence between White and Black patients explained by covariates, and then calculate the contribution each covariate to the disparity in medication adherence by race. Pre-specified covariates will include those already collected during the study (demographics, clinical factors, medication use, health care utilization, perceived discrimination, attitudinal factors, and behavioral factors). **UCD will share a de-identified data set with one of our subcontractors (Kaiser Permanente Mid-Atlantic States), and they will conduct these analyses on site.**

G. Summarize Knowledge to be Gained:

Potential benefits of the proposed research to the subjects and others: Benefit from participating in the research cannot be claimed. If, however, the intervention being studied is effective, subjects randomized to the experimental group may experience improvements in blood pressure control and other parameters dependent on uptake of self-management skills. Given the magnitude of the problem of race-based health disparities, the potential of the proposed intervention to reduce these disparities, and the minimal risk to subjects, we are of the opinion that the risk-benefit ratio for this study is favorable.

Importance of the knowledge to be gained: Our preliminary data suggest the intervention will be effective. The current study is designed to generate knowledge that will justify widespread adoption if effectiveness is demonstrated. Because virtually no interventions to improve relationships between physicians and minority patients have been reported, a positive result of this study might have widespread clinical applicability. We also seek data on the potential for this social psychology intervention to have direct effect on blood pressure.

IN SUMMARY, our proposed study moves beyond documentation of race-based health disparities and towards an effective intervention. We focus on a medical condition – hypertension – that is arguably the single greatest contributor to mortality disparities for African

Americans. Our pilot data suggest a values-affirmation intervention improves patient-provider communication, which in turn improves subsequent medication adherence. Our pilot studies, however, leave gaps in knowledge that we propose to answer to determine the effectiveness of using values affirmation as a means of reducing racial disparities in health care. If successful, this study will be the first to provide evidence for a low-resource intervention that has the potential to substantially reduce healthcare disparities across a wide range of health care conditions and populations.

H. References:

1. Go AS, Mozaffarian D, Roger VL, Benjamin EJ, Berry JD, Borden WB, Bravata DM, Dai S, Ford ES, Fox CS, Franco S, Fullerton HJ, Gillespie C, Hailpern SM, Heit JA, Howard VJ, Huffman MD, Kissela BM, Kittner SJ, Lackland DT, Lichtman JH, Lisabeth LD, Magid D, Marcus GM, Marelli A, Matchar DB, McGuire DK, Mohler ER, Moy CS, Mussolino ME, Nichol G, Paynter NP, Schreiner PJ, Sorlie PD, Stein J, Turan TN, Virani SS, Wong ND, Woo D, Turner MB, Committee obotAHAS and Subcommittee SS. Heart Disease and Stroke Statistics—2013 Update A Report From the American Heart Association. *Circulation*. 2012.
2. Centers for Disease Control and Prevention. Vital signs: prevalence, treatment, and control of hypertension--United States, 1999-2002 and 2005-2008. *MMWR Morb Mortal Wkly Rep*. 2011;60:103-8.
3. Yoon SS, Gu Q, Nwankwo T, Wright JD, Hong Y and Burt V. Trends in Blood Pressure Among Adults With Hypertension: United States, 2003 to 2012. *Hypertension*. 2014.
4. Howard G, Lackland DT, Kleindorfer DO, Kissela BM, Moy CS, Judd SE, Safford MM, Cushman M, Glasser SP and Howard VJ. Racial differences in the impact of elevated systolic blood pressure on stroke risk. *JAMA Intern Med*. 2013;173:46-51.
5. Young JH, Ng D, Ibe C, Weeks K, Brotman DJ, Dy SM, Brancati FL, Levine DM and Klag MJ. Access to Care, Treatment Ambivalence, Medication Nonadherence, and Long-Term Mortality Among Severely Hypertensive African Americans: A Prospective Cohort Study. *Journal of Clinical Hypertension*. 2015;17:614-621.
6. Bosworth HB and Oddone EZ. A model of psychosocial and cultural antecedents of blood pressure control. *Journal of the National Medical Association*. 2002;94:236-48.
7. Kressin NR, Orner MB, Manze M, Glickman ME and Berlowitz D. Understanding contributors to racial disparities in blood pressure control. *Circ Cardiovasc Qual Outcomes*. 2010;3:173-80.
8. Institute of Medicine (US) Committee on Public Health Priorities to Reduce and Control Hypertension. Interventions Directed at Individuals with Hypertension. *A Population-Based Policy and Systems Change Approach to Prevent and Control Hypertension*. 2010;2016.
9. Dragomir A, Cote R, Roy L, Blais L, Lalonde L, Berard A and Perreault S. Impact of adherence to antihypertensive agents on clinical outcomes and hospitalization costs. *Med Care*. 2010;48:418-25.
10. Elliott WJ. Improving outcomes in hypertensive patients: focus on adherence and persistence with antihypertensive therapy. *J Clin Hypertens (Greenwich)*. 2009;11:376-82.
11. World Health Organization. Adherence to long-term therapies: Evidence for action. 2003.
12. Osterberg L and Blaschke T. Adherence to Medication. *N Engl J Med*. 2005;353:487-497.
13. Roebuck MC, Liberman JN, Gemmill-Toyama M and Brennan TA. Medication adherence leads to lower health care use and costs despite increased drug spending. *Health affairs (Project Hope)*. 2011;30:91-9.
14. Bramley T, Gerbino P, Nightengale B and Frech-Tamas F. Relationship of blood pressure control to adherence with antihypertensive monotherapy in 13 managed care organizations. *J Manag Care Pharm*. 2006;12:239-45.
15. Mazzaglia G, Ambrosioni E, Alacqua M, Filippi A, Sessa E, Immordino V, Borghi C, Brignoli O, Caputi AP, Cricelli C and Mantovani LG. Adherence to antihypertensive medications and cardiovascular morbidity among newly diagnosed hypertensive patients. *Circulation*. 2009;120:1598-605.

16. Lee JK, Grace KA and Taylor AJ. Effect of a pharmacy care program on medication adherence and persistence, blood pressure, and low-density lipoprotein cholesterol: a randomized controlled trial. *Jama*. 2006;296:2563-71.
17. Lewis LM, Ogedegbe C and Ogedegbe G. Enhancing adherence of antihypertensive regimens in hypertensive African-Americans: current and future prospects. *Expert review of cardiovascular therapy*. 2012;10:1375-80.
18. Kressin N, Wang F, Long J, Bokhour B, Orner M, Rothendler J, Clark C, Reddy S, Kozak W, Kroupa L and Berlowitz D. Hypertensive Patients' Race, Health Beliefs, Process of Care, and Medication Adherence. *Journal of General Internal Medicine*. 2007;22:768-774.
19. Bosworth HB, Powers B, Grubber JM, Thorpe CT, Olsen MK, Orr M and Oddone EZ. Racial Differences in Blood Pressure Control: Potential Explanatory Factors. *Journal of General Internal Medicine*. 2008;23:692-698.
20. Blair IV, Steiner JF, Hanratty R, Price DW, Fairclough DL, Daugherty SL, Bronsert M, Magid DJ and Havranek EP. An investigation of associations between clinicians' ethnic or racial bias and hypertension treatment, medication adherence and blood pressure control. *Journal of General Internal Medicine*. 2014;29:987-95.
21. Burgess DJ, Warren J, Phelan S, Dovidio J and van Ryn M. Stereotype threat and health disparities: what medical educators and future physicians need to know. *Journal of General Internal Medicine*. 2010;25 Suppl 2:S169-77.
22. Zuckerman RB, Tinsley LJ, Hawk H and Cohen B. Perceived Reactions to Race and Health Status in the Massachusetts Behavioral Risk Factor Surveillance System Survey. *Ethnic Dis*. 2012;22:492-496.
23. Chung BW, Meldrum M, Jones F, Brown A and Jones L. Perceived Sources of Stress and Resilience in Men in an African American Community. *Prog Comm Hlth Partn*. 2014;8:441-451.
24. Sorkin DH, Ngo-Metzger Q and De Alba I. Racial/Ethnic Discrimination in Health Care: Impact on Perceived Quality of Care. *Journal of General Internal Medicine*. 2010;25:390-396.
25. Dolezsar CM, McGrath JJ, Herzig AJM and Miller SB. Perceived Racial Discrimination and Hypertension: A Comprehensive Systematic Review. *Health Psychology*. 2014;33:20-34.
26. Anderson KF. Diagnosing Discrimination: Stress from Perceived Racism and the Mental and Physical Health Effects. *Sociol Inq*. 2013;83:55-81.
27. Ikram UZ, Snijder MB, Fassaert TJL, Schene AH, Kunst AE and Stronks K. The contribution of perceived ethnic discrimination to the prevalence of depression. *European Journal of Public Health*. 2015;25:243-248.
28. LeBron AMW, Valerio MA, Kieffer E, Sinco B, Rosland AM, Hawkins J, Espitia N, Palmisano G and Spencer M. Everyday Discrimination, Diabetes-Related Distress, and Depressive Symptoms Among African Americans and Latinos with Diabetes. *J Immigr Minor Healt*. 2014;16:1208-1216.
29. Thames AD, Hinkin CH, Byrd DA, Bilder RM, Duff KJ, Mindt MR, Arentoft A and Streiff V. Effects of Stereotype Threat, Perceived Discrimination, and Examiner Race on Neuropsychological Performance: Simple as Black and White? *Journal of the International Neuropsychological Society*. 2013;19:583-593.
30. Sims M, Diez-Roux AV, Dudley A, Gebreab S, Wyatt SB, Bruce MA, James SA, Robinson JC, Williams DR and Taylor HA. Perceived Discrimination and Hypertension Among African Americans in the Jackson Heart Study. *American Journal of Public Health*. 2012;102:S258-S265.
31. Harris R, Cormack D, Tobias M, Yeh LC, Talamaivao N, Minster J and Timutimu R. The pervasive effects of racism: Experiences of racial discrimination in New Zealand over time and associations with multiple health domains. *Social Science & Medicine*. 2012;74:408-415.
32. Cuffee YL, Hargraves JL, Rosal M, Briesacher BA, Schoenthaler A, Person S, Hullett S and Allison J. Reported Racial Discrimination, Trust in Physicians, and Medication Adherence Among Inner-City African Americans With Hypertension. *American Journal of Public Health*. 2013;103:E55-E62.
33. Hausmann LRM, Kressin NR, Hanusa BH and Ibrahim SA. Perceived Racial Discrimination in Health Care and Its Association with Patients' Healthcare Experiences: Does the Measure Matter? *Ethnic Dis*. 2010;20:40-47.

34. Van Houtven CH, Voils CI, Oddone EZ, Weinfurt KP, Friedman JY, Schulman KA and Bosworth HB. Perceived discrimination and reported delay of pharmacy prescriptions and medical tests. *Journal of General Internal Medicine*. 2005;20:578-583.
35. Casagrande SS, Gary TL, LaVeist TA, Gaskin DJ and Cooper LA. Perceived discrimination and adherence to medical care in a racially integrated community. *Journal of General Internal Medicine*. 2007;22:389-395.
36. Cuevas AG, O'Brien K and Saha S. African American Experiences in Healthcare: "I Always Feel Like I'm Getting Skipped Over". *Health psychology : official journal of the Division of Health Psychology, American Psychological Association*. 2016.
37. Forsyth J, Schoenthaler A, Chaplin WF, Ogedegbe G and Ravenell J. Perceived discrimination and medication adherence in black hypertensive patients: the role of stress and depression. *Psychosomatic medicine*. 2014;76:229-36.
38. Becker G and Newsom E. Socioeconomic status and dissatisfaction with health care among chronically ill African Americans. *Am J Public Health*. 2003;93:742-8.
39. Hatzfeld JJ, Cody-Connor C, Whitaker VB and Gaston-Johansson F. African-American perceptions of health disparities: a qualitative analysis. *Journal of National Black Nurses' Association : JNBNA*. 2008;19:34-41.
40. Gaston-Johansson F, Hill-Briggs F, Oguntomilade L, Bradley V and Mason P. Patient perspectives on disparities in healthcare from African-American, Asian, Hispanic, and Native American samples including a secondary analysis of the Institute of Medicine focus group data. *Journal of National Black Nurses' Association : JNBNA*. 2007;18:43-52.
41. Schmader T and Johns M. Converging evidence that stereotype threat reduces working memory capacity. *J Pers Soc Psychol*. 2003;85:440-52.
42. Blascovich J, Spencer SJ, Quinn D and Steele C. African Americans and high blood pressure: the role of stereotype threat. *Psychol Sci*. 2001;12:225-9.
43. Institute of Medicine. Supporting Patients' Decision-Making Abilities and Preferences. *Improving the Quality of Health Care for Mental and Substance-Use Conditions: Quality Chasm Series Institute of Medicine (US) Committee on Crossing the Quality Chasm: Adaptation to Mental Health and Addictive Disorders*. 2006.
44. Burgess DJ, Ding YM, Hargreaves M, van Ryn M and Phelan S. The association between perceived discrimination and underutilization of needed medical and mental health care in a multi-ethnic community sample. *Journal of health care for the poor and underserved*. 2008;19:894-911.
45. Cohen GL and Sherman DK. The psychology of change: self-affirmation and social psychological intervention. *Annual review of psychology*. 2014;65:333-71.
46. Sherman DK and Cohen GL. The Psychology of Self-defense: Self-Affirmation Theory. In: P. Z. Mark, ed. *Advances in Experimental Social Psychology*: Academic Press; 2006(38): 183-242.
47. Steele CM and Aronson J. Stereotype threat and the intellectual test performance of African Americans. *Journal of Personality and Social Psychology*. 1995;69:797-811.
48. Shnabel N, Purdie-Vaughns V, Cook JE, Garcia J and Cohen GL. Demystifying Values-Affirmation Interventions: Writing About Social Belonging Is a Key to Buffering Against Identity Threat. *Pers Soc Psychol B*. 2013;39:663-676.
49. Cohen GL, Garcia J, Apfel N and Master A. Reducing the racial achievement gap: a social-psychological intervention. *Science (New York, NY)*. 2006;313:1307-10.
50. Cohen GL, Garcia J, Purdie-Vaughns V, Apfel N and Brzustoski P. Recursive processes in self-affirmation: intervening to close the minority achievement gap. *Science (New York, NY)*. 2009;324:400-3.
51. Miyake A, Kost-Smith LE, Finkelstein ND, Pollock SJ, Cohen GL and Ito TA. Reducing the gender achievement gap in college science: a classroom study of values affirmation. *Science (New York, NY)*. 2010;330:1234-7.
52. Martens A, Johns M, Greenberg J and Schimel J. Combating stereotype threat: The effect of self-affirmation on women's intellectual performance. *Journal of Experimental Social Psychology*. 2006;42:236-243.
53. Creswell JD, Welch WT, Taylor SE, Sherman DK, Gruenewald TL and Mann T. Affirmation of personal values buffers neuroendocrine and psychological stress responses. *Psychological Science*. 2005;16:846-851.

54. Walton GM and Cohen GL. A brief social-belonging intervention improves academic and health outcomes of minority students. *Science (New York, NY)*. 2011;331:1447-51.

55. Yeager DS and Walton GM. Social-Psychological Interventions in Education: They're Not Magic. *Review of Educational Research*. 2011;81:267-301.

56. Gruman J, Rovner MH, French ME, Jeffress D, Sofaer S, Shaller D and Prager DJ. From patient education to patient engagement: implications for the field of patient education. *Patient Education and Counseling*. 2010;78:350-6.

57. Greer TM, Brondolo E and Brown P. Systemic Racism Moderates Effects of Provider Racial Biases on Adherence to Hypertension Treatment for African Americans. *Health Psychology*. 2014;33:35-42.

58. Hagiwara N, Penner LA, Gonzalez R, Eggly S, Dovidio JF, Gaertner SL, West T and Albrecht TL. Racial attitudes, physician-patient talk time ratio, and adherence in racially discordant medical interactions. *Social Science & Medicine*. 2013;87:123-131.

59. Lambert-Kerzner A, Havranek EP, Plomondon ME, Fagan KM, McCreight MS, Fehling KB, Williams DJ, Hamilton AB, Albright K, Blatchford PJ, Mihalko-Corbitt R, Bryson CL, Bosworth HB, Kirshner MA, Del Giacco EJ and Ho PM. Perspectives of patients on factors relating to adherence to post-acute coronary syndrome medical regimens. *Patient Prefer Adher*. 2015;9:1053-1059.

60. Blair IV, Steiner JF, Fairclough DL, Hanratty R, Price DW, Hirsh HK, Wright LA, Bronsert M, Karimkhani E, Magid DJ and Havranek EP. Clinicians' implicit ethnic/racial bias and perceptions of care among Black and Latino patients. *Annals of family medicine*. 2013;11:43-52.

61. Cooper LA, Roter DL, Carson KA, Beach MC, Sabin JA, Greenwald AG and Inui TS. The Associations of Clinicians' Implicit Attitudes About Race With Medical Visit Communication and Patient Ratings of Interpersonal Care. *American Journal of Public Health*. 2012;102:979-987.

62. Shavers VL, Fagan P, Jones D, Klein WMP, Boyington J, Moten C and Rorie E. The State of Research on Racial/Ethnic Discrimination in The Receipt of Health Care. *American Journal of Public Health*. 2012;102:953-966.

63. Alexander J, Hearld L and Mittler JN. Patient–Physician Role Relationships and Patient Activation: The Moderating Effects of Race and Ethnicity. *Medical Care Research and Review*. 2014.

64. Parchman ML, Zeber JE and Palmer RF. Participatory Decision Making, Patient Activation, Medication Adherence, and Intermediate Clinical Outcomes in Type 2 Diabetes: A STARNet Study. *Annals of family medicine*. 2010;8:410-417.

65. Roumie CL, Wallston KA, Greevy R, Elasy TA, Stone WJ, Liu X, Dittus RS and Speroff T. Patient self reported levels of patient centered care and activation predicts medication adherence among veterans with hypertension. *Journal of General Internal Medicine*. 2008;23:359-359.

66. Smedley BD, Stith AY and Nelson AR. *Unequal Treatment: Confronting Racial and Ethnic Disparities in Health Care*. Washington (DC): National Academies Press (US); 2003.

67. Blair IV, Steiner JF and Havranek EP. Unconscious (implicit) bias and health disparities: where do we go from here? *The Permanente journal*. 2011;15:71-8.

68. Blair IV. The malleability of automatic stereotypes and prejudice. *Personality and Social Psychology Review*. 2002;6:242-261.

69. Blair IV, Ma JE and Lenton AP. Imagining Stereotypes Away: The Moderation of Implicit Stereotypes Through Mental Imagery. *Journal of Personality & Social Psychology*. 2001;81:828-841.

70. Blair IV, Havranek EP, Price DW, Hanratty R, Fairclough DL, Farley T, Hirsh HK and Steiner JF. Assessment of biases against Latinos and African Americans among primary care providers and community members. *Am J Public Health*. 2013;103:92-8.

71. Daugherty SL, Blair IV, Havranek EP, Dickinson M, Bronsert M, Karimkhani E, Main DS and Masoudi FA. Gender bias among cardiology physicians. Paper presented at: AHA Quality of Care and Outcomes Research Conference; 2014; Baltimore, MD.

72. Sabin JA and Greenwald AG. The influence of implicit bias on treatment recommendations for 4 common pediatric conditions: pain, urinary tract infection, attention deficit hyperactivity disorder, and asthma. *Am J Public Health*. 2012;102:988-95.

73. Horvat L, Horey D, Romios P and Kis-Rigo J. Cultural competence education for health professionals. *Cochrane database of systematic reviews (Online)*. 2014;5:CD009405.

74. Truong M, Paradies Y and Priest N. Interventions to improve cultural competency in healthcare: a systematic review of reviews. *BMC Health Serv Res*. 2014;14:99.
75. Stone J and Moskowitz GB. Non-conscious bias in medical decision making: what can be done to reduce it? *Medical education*. 2011;45:768-76.
76. Burgess D, van Ryn M, Dovidio J and Saha S. Reducing racial bias among health care providers: lessons from social-cognitive psychology. *J Gen Intern Med*. 2007;22:882-7.
77. Beune EJ, Haafkens JA and Bindels PJ. Barriers and enablers in the implementation of a provider-based intervention to stimulate culturally appropriate hypertension education. *Patient Educ Couns*. 2011;82:74-80.
78. Roter D and Larson S. The Roter interaction analysis system (RIAS): utility and flexibility for analysis of medical interactions. *Patient Education and Counseling*. 2002;46:243-51.
79. Havranek EP, Hanratty R, Tate C, Dickinson LM, Steiner JF, Cohen G and Blair IA. The effect of values affirmation on race-discordant patient-provider communication. *Arch Intern Med*. 2012;172:1662-7.
80. Viswanathan M, Golin CE, Jones CD, Ashok M, Blalock SJ, Wines RC, Coker-Schwimmer EJ, Rosen DL, Sista P and Lohr KN. Interventions to improve adherence to self-administered medications for chronic diseases in the United States: a systematic review. *Annals of Internal Medicine*. 2012;157:785-95.
81. Gaglio B, Shoup JA and Glasgow RE. The RE-AIM framework: a systematic review of use over time. *American Journal of Public Health*. 2013;103:e38-46.
82. Glasgow RE, Dickinson P, Fisher L, Christiansen S, Toobert DJ, Bender BG, Dickinson LM, Jortberg B and Estabrooks PA. Use of RE-AIM to develop a multi-media facilitation tool for the patient-centered medical home. *Implementation science : IS*. 2011;6:118.
83. Glasgow RE, Vogt TM and Boles SM. Evaluating the public health impact of health promotion interventions: the RE-AIM framework. *American Journal of Public Health*. 1999;89:1322-7.
84. Steiner JF, Koepsell TD, Fihn SD and Inui TS. A general method of compliance assessment using centralized pharmacy records. Description and validation. *Medical Care*. 1988;26:814-23.
85. Steiner JF and Prochazka AV. The assessment of refill compliance using pharmacy records: methods, validity, and applications. *Journal of Clinical Epidemiology*. 1997;50:105-16.
86. Choo PW, Rand CS, Inui TS, Lee ML, Cain E, Cordeiro-Breault M, Canning C and Platt R. Validation of patient reports, automated pharmacy records, and pill counts with electronic monitoring of adherence to antihypertensive therapy. *Medical Care*. 1999;37:846-57.
87. Farley J, Hines S, Musk A, Ferrus S and Tepper V. Assessment of adherence to antiviral therapy in HIV-infected children using the Medication Event Monitoring System, pharmacy refill, provider assessment, caregiver self-report, and appointment keeping. *Journal of acquired immune deficiency syndromes (1999)*. 2003;33:211-8.
88. Ho PM, Magid DJ, Shetterly SM, Olson KL, Maddox TM, Peterson PN, Masoudi FA and Rumsfeld JS. Medication nonadherence is associated with a broad range of adverse outcomes in patients with coronary artery disease. *American Heart Journal*. 2008;155:772-779.
89. Ho PM, Rumsfeld JS, Masoudi FA, McClure DL, Plomondon ME, Steiner JF and Magid DJ. Effect of Medication Nonadherence on Hospitalization and Mortality Among Patients With Diabetes Mellitus. *Arch Intern Med*. 2006;166:1836-1841.
90. Daugherty SL, Powers JD, Magid DJ, Masoudi FA, Margolis KL, O'Connor PJ, Schmittdiel JA and Ho PM. The association between medication adherence and treatment intensification with blood pressure control in resistant hypertension. *Hypertension*. 2012;60:303-9.
91. Daugherty SL, Powers JD, Magid DJ, Tavel HM, Masoudi FA, Margolis KL, O'Connor PJ, Selby JV and Ho PM. Incidence and prognosis of resistant hypertension in hypertensive patients. *Circulation*. 2012;125:1635-42.
92. Steiner JF. Self-reported Adherence Measures What Do They Assess and How Should We Use Them? *Medical Care*. 2012;50:1011-1012.
93. Steiner JF. Rethinking Adherence. *Annals of Internal Medicine*. 2012;157:580-+.
94. Steiner JF, Ho PM, Beaty BL, Dickinson LM, Hanratty R, Zeng C, Tavel HM, Havranek EP, Davidson AJ, Magid DJ and Estacio RO. Sociodemographic and Clinical Characteristics Are Not Clinically Useful Predictors of Refill Adherence in Patients With Hypertension. *Circ-Cardiovasc Qual*. 2009;2:451-457.

95. Steiner JF and Gardner EM. Assessing medication adherence from pharmacy records. *Pharmacoepidemiology and Drug Safety*. 2006;15:575-577.
96. Fitzgerald AA, Peterson PN, Magid DJ, Masoudi FA, Allen L, Clarke CL, Ho M, Shetterly S, Powers D and Havranek EP. Adherence to Evidence-Based Pharmacotherapy Reduces the Risk of Death and Rehospitalization in Patients With Heart Failure. *Journal of the American College of Cardiology*. 2009;53:A378-A378.
97. Morgan A, Masoudi FA, Havranek E, Jones P, Peterson PN, Spertus JA and Rumsfeld JS. Difficulty with medication adherence is associated with worse health status in heart failure patients. *Circulation*. 2004;109:E242-E242.
98. Voils CI, Maciejewski ML, Hoyle RH, Reeve BB, Gallagher P, Bryson CL and Yancy WS. Initial Validation of a Self-Report Measure of the Extent of and Reasons for Medication Nonadherence. *Medical Care*. 2012;50:1013-1019.
99. Voils CI, Maciejewski M, Hoyle R, Reeve B, Gallagher P and Yancy W. Developing a Theoretically Informed Measure to Detect and Address Self-Reported Medication Nonadherence. *Annals of Behavioral Medicine*. 2012;43:S178-S178.
100. James PA, Oparil S, Carter BL, Cushman WC, Dennison-Himmelfarb C, Handler J, Lackland DT, LeFevre ML, MacKenzie TD, Ogedegbe O, Smith SC, Jr., Svetkey LP, Taler SJ, Townsend RR, Wright JT, Jr., Narva AS and Ortiz E. 2014 evidence-based guideline for the management of high blood pressure in adults: report from the panel members appointed to the Eighth Joint National Committee (JNC 8). *Jama*. 2014;311:507-20.
101. James PA, Oparil S, Carter BL and et al. 2014 evidence-based guideline for the management of high blood pressure in adults: Report from the panel members appointed to the eighth joint national committee (jnc 8). *JAMA*. 2014;311:507-520.
102. Vigen R, Shetterly S, Magid DJ, O'Connor PJ, Margolis KL, Schmittdiel J and Ho PM. A comparison between antihypertensive medication adherence and treatment intensification as potential clinical performance measures. *Circ Cardiovasc Qual Outcomes*. 2012;5:276-82.
103. Ho PM, Magid DJ, Shetterly SM, Olson KL, Peterson PN, Masoudi FA and Rumsfeld JS. Importance of therapy intensification and medication nonadherence for blood pressure control in patients with coronary disease. *Arch Intern Med*. 2008;168:271-6.
104. Rose AJ, Berlowitz DR, Orner MB and Kressin NR. Understanding Uncontrolled Hypertension: Is It the Patient or the Provider? *The Journal of Clinical Hypertension*. 2007;9:937-943.
105. Maddox TM, Ross C, Tavel HM, Lyons EE, Tillquist M, Ho PM, Rumsfeld JS, Margolis KL, O'Connor PJ, Selby JV and Magid DJ. Blood pressure trajectories and associations with treatment intensification, medication adherence, and outcomes among newly diagnosed coronary artery disease patients. *Circ Cardiovasc Qual Outcomes*. 2010;3:347-57.
106. Rose AJ, Berlowitz DR, Manze M, Orner MB and Kressin NR. Comparing Methods of Measuring Treatment Intensification in Hypertension Care. *Circ Cardiovasc Qual Outcomes*. 2009;2:385-391.
107. Blair IV, Steiner JF, Hanratty R, Price DW, Fairclough DL, Daugherty SL, Bronsert M, Magid DJ and Havranek EP. An Investigation of Associations Between Clinicians' Ethnic or Racial Bias and Hypertension Treatment, Medication Adherence and Blood Pressure Control. *Journal of General Internal Medicine*. 2014;29:987-995.
108. Gabow P, Eisert S and Wright R. Denver Health: a model for the integration of a public hospital and community health centers. *Annals of Internal Medicine*. 2003;138:143-9.
109. Nuzum R, McCarthy D, Gauthier A and Beck C. Denver Health: A High-Performance Public Health Care System. *The Commonwealth Fund Reports*. 2007.
110. Raebel MA, Carroll NM, Ellis JL, Schroeder EB and Bayliss EA. Importance of including early nonadherence in estimations of medication adherence. *Annals of Pharmacotherapy*. 2011;45:1053-60.
111. Magid DJ, Olson KL, Billups SJ, Wagner NM, Lyons EE and Kroner BA. A Pharmacist-Led, American Heart Association Heart360 Web-Enabled Home Blood Pressure Monitoring Program. *Circ-Cardiovasc Qual*. 2013;6:157-+.
112. Nichols GA, Desai J, Lafata JE, Lawrence JM, O'Connor PJ, Pathak RD, Raebel MA, Reid RJ, Selby JV, Silverman BG, Steiner JF, Stewart WF, Vupputuri S, Waitzfelder B and Grp S-DS. Construction of a Multisite DataLink Using Electronic Health Records for the Identification,

Surveillance, Prevention, and Management of Diabetes Mellitus: The SUPREME-DM Project. *Preventing chronic disease*. 2012;9.

113. Daugherty SL, Blair IV, Havranek EP, Furniss A, Dickinson M, Main DS, Karimkhani E and Masoudi FA. Genders attitudes and use of angiography among cardiologists'. Paper presented at: AHA Scientific Sessions; 2015; Orlando, FL.

114. Hanratty R, Chonchol M, Havranek EP, Powers JD, Dickinson LM, Ho PM, Magid DJ and Steiner JF. Relationship between Blood Pressure and Incident Chronic Kidney Disease in Hypertensive Patients. *Clinical Journal of the American Society of Nephrology*. 2011;6:2605-2611.

115. Blair IV, Judd CM, Havranek EP and Steiner JF. Using Community Data to Test the Discriminate Validity of Ethnic/Racial Group IATs. *Zeitschrift fur Psychologie*. 2010;218.

116. Hanratty R, Estacio RO, Dickinson LM, Chandramouli V, Steiner JF, Havranek EP and Latino Using Cardio Health Actions to Reduce Risk study i. Testing electronic algorithms to create disease registries in a safety net system. *Journal of health care for the poor and underserved*. 2008;19:452-65.

117. Sandhu A, Ho PM, Asche S, Magid DJ, Margolis KL, Sperl-Hillen J, Rush B, Price DW, Ekstrom H, Tavel H, Godlevsky O and O'Connor PJ. Recidivism to uncontrolled blood pressure in patients with previously controlled hypertension. *Am Heart J*. 2015;169:791-7.

118. Schroeder EB, Hanratty R, Beaty BL, Bayliss EA, Havranek EP and Steiner JF. Simultaneous Control of Diabetes Mellitus, Hypertension, and Hyperlipidemia in 2 Health Systems. *Circulation: Cardiovascular Quality and Outcomes*. 2012.

119. Brondolo E, Kelly KP, Coakley V, Gordon T, Thompson S, Levy E, Cassells A, Tobin JN, Sweeney M and Contrada RJ. The Perceived Ethnic Discrimination Questionnaire: Development and Preliminary Validation of a Community Version1. *Journal of Applied Social Psychology*. 2005;35:335-365.

120. Hibbard JH, Mahoney ER, Stockard J and Tusler M. Development and testing of a short form of the patient activation measure. *Health Services Research*. 2005;40:1918-30.

121. Taylor SD, Bagozzi RP and Gaither CA. Decision making and effort in the self-regulation of hypertension: testing two competing theories. *British journal of health psychology*. 2005;10:505-30.

122. van Ryn M, Hardeman R, Phelan S, PhD DB, Dovidio J, Herrin J, Burke S, Nelson D, Perry S, Yeazel M and Przedworski J. Medical School Experiences Associated with Change in Implicit Racial Bias Among 3547 Students: A Medical Student CHANGES Study Report. *Journal of General Internal Medicine*. 2015:1-9.

123. Dovidio JF, Penner LA, Albrecht TL, Norton WE, Gaertner SL and Shelton JN. Disparities and distrust: The implications of psychological processes for understanding racial disparities in health and health care. *Social Science & Medicine*. 2008;67:478-486.

124. Blanchard J, Nayar S and Lurie N. Patient-provider and patient-staff racial concordance and perceptions of mistreatment in the health care setting. *Journal of general internal medicine*. 2007;22:1184-9.

125. Johnson RL, Saha S, Arbelaez JJ, Beach MC and Cooper LA. Racial and ethnic differences in patient perceptions of bias and cultural competence in health care. *Journal of general internal medicine*. 2004;19:101-10.

126. Lyles CR, Karter AJ, Young BA, Spigner C, Grembowski D, Schillinger D and Adler N. Provider factors and patient-reported healthcare discrimination in the Diabetes Study of California (DISTANCE). *Patient education and counseling*. 2011;85:e216-24.

127. Lyles CR, Karter AJ, Young BA, Spigner C, Grembowski D, Schillinger D and Adler N. Patient-reported racial/ethnic healthcare provider discrimination and medication intensification in the Diabetes Study of Northern California (DISTANCE). *Journal of general internal medicine*. 2011;26:1138-44.

128. Peek ME, Wagner J, Tang H, Baker DC and Chin MH. Self-reported racial discrimination in health care and diabetes outcomes. *Medical care*. 2011;49:618-25.

129. Hibbard JH, Mahoney ER, Stock R and Tusler M. Do increases in patient activation result in improved self-management behaviors? *Health Services Research*. 2007;42:1443-63.

130. Hibbard JH, Greene J, Becker ER, Roblin D, Painter MW, Perez DJ, Burbank-Schmitt E and Tusler M. Racial/ethnic disparities and consumer activation in health. *Health Affairs*. 2008;27:1442-53.

131. Perugini M and Bagozzi RP. The role of desires and anticipated emotions in goal-directed behaviours: broadening and deepening the theory of planned behaviour. *The British journal of social psychology / the British Psychological Society*. 2001;40:79-98.
132. Ajzen I. The theory of planned behavior. *Organizational Behavior and Human Decision Processes*. 1991;50:179-211.
133. Barr DA. Race/ethnicity and patient satisfaction - Using the appropriate method to test for perceived differences in care. *Journal of General Internal Medicine*. 2004;19:937-943.
134. Greenwald AG, McGhee DE and Schwartz JLK. Measuring individual differences in implicit cognition: The implicit association test. *Journal of Personality and Social Psychology*. 1998;74:1464-1480.
135. Greenwald AG, Nosek BA and Banaji MR. Understanding and Using the Implicit Association Test: I. An Improved Scoring Algorithm. *Journal of Personality & Social Psychology*. 2003;85:197-216.
136. Nosek BA, Greenwald AG and Banaji MR. Understanding and Using the Implicit Association Test: II. Method Variables and Construct Validity. *Pers Soc Psychol Bull*. 2005;31:166-180.
137. Nosek BA and Smyth FL. A Multitrait-Multimethod Validation of the Implicit Association Test: Implicit and Explicit Attitudes Are Related but Distinct Constructs. *Experimental Psychology*. 2007;54:14-29.
138. Cunningham WA, Preacher KJ and Banaji MR. Implicit Attitude Measures: Consistency, Stability, and Convergent Validity. *Psychological Science*. 2001;12:163-170.
139. Lane K, Banaji M, Nosek B and Greenwald A. *Understanding and using the Implicit Association Test: IV: What we know (so far) about the method. In: Implicit Measures of Attitudes.* New York, NY: Guilford Press; 2007.
140. Greenwald AG, Poehlman TA, Uhlmann EL and Banaji MR. Understanding and Using the Implicit Association Test: III. Meta-Analysis of Predictive Validity. *Journal of Personality and Social Psychology*. 2009;97:17-41.
141. Dovidio JF, Kawakami K and Gaertner SL. Implicit and explicit prejudice and interracial interaction. *Journal of Personality and Social Psychology*. 2002;82:62-68.
142. Brannon TN and Walton GM. Enacting Cultural Interests: How Intergroup Contact Reduces Prejudice by Sparking Interest in an Out-Group's Culture. *Psychological Science*. 2013;24:1947-1957.
143. Saha S, Guiton G, Wimmers PF and Wilkerson L. Student body racial and ethnic composition and diversity-related outcomes in US medical schools. *JAMA-J Am Med Assoc*. 2008;300:1135-1145.
144. Ross TR, Ng D, Brown JS, Pardee R, Hornbrook MC, Hart G and Steiner JF. The HMO Research Network Virtual Data Warehouse: A Public Data Model to Support Collaboration. *EGEMS (Washington, DC)*. 2014;2:1049.
145. Krousel-Wood M, Thomas S, Muntner P and Morisky D. Medication adherence: a key factor in achieving blood pressure control and good clinical outcomes in hypertensive patients. *Current Opinion in Cardiology*. 2004;19:357-362.
146. Ho PM, Bryson CL and Rumsfeld JS. Medication Adherence: Its Importance in Cardiovascular Outcomes. *Circulation*. 2009;119:3028-3035.
147. Hayes AF. Beyond Baron and Kenny: Statistical Mediation Analysis in the New Millennium. *Communication Monographs*. 2009;76: 408-420.
148. Hedeker D and Gibbons R. *Longitudinal data analysis*. Hoboken, New Jersey: John Wiley & Sons; 2006.
