# Supplementary material for: Effect of Values Affirmation on Reducing Racial Differences in Adherence to Hypertension Medication: The HYVALUE Randomized Clinical Trial
Source: JAMA Netw Open. 2021 Dec 16;4(12):e2139533. doi: 10.1001/jamanetworkopen.2021.39533 (PMC8678693; doi:10.1001/jamanetworkopen.2021.39533)
Supplement: Supplement 2. — eMethods 1. Fidelity of Intervention eMethods 2. Clinician-Level Clustering eTable. Characteristics of the Eligible, Not Enrolled to the Enrolled Study Population [file jamanetwopen-e2139533-s002.pdf]

Supplementary Online Content

Daugherty SL, Helmkamp L, Vupputuri S, et al. Effect of values affirmation on reducing racial differences in adherence to hypertension medication: the HYVALUE randomized clinical trial. *JAMA Netw Open.* 2021;4(12):e2139533. doi:10.1001/jamanetworkopen.2021.39533

eMethods 1. Fidelity of Intervention
eMethods 2. Clinician-Level Clustering
eTable. Characteristics of the Eligible, Not Enrolled to the Enrolled Study Population

This supplementary material has been provided by the authors to give readers additional information about their work.

© 2021 Daugherty SL et al. *JAMA Network Open.*

eMethods 1. Fidelity of Intervention

Fidelity to Instructions and Self-Affirmation

Two primary coders independently read and transcribed the written text. The text was coded using published criteria to assess *fidelity to instructions* ('Did the patient identify at least one value from the list?') and *self-affirmation* ('Did the patient write about the value for themselves?' and 'Did the patient express either the importance of the value or write about being 'good' in the valued domain?'). Each primary coder trained on 48 entries that had been previously coded by the project leads (S.L.D, J.A.M) before independently coding additional entries. Agreement between the coders was high (kappa = 0.75). Entries with unclear meanings were flagged and independently coded by a second blinded coder (J.A.M). Disagreements were discussed, and a final designation was determined.

Fit of the Intervention:

To understand the salience of the values chosen (i.e., fit of the intervention to population), patients in the intervention arm were asked to indicate if they agreed with four statements concerning their selected values: 1) "These values have influenced my life", 2) "In general, I try to live up to these values", 3) "These values are an important part of who I am", and 4) "I care about these values". In the control arm, patients were asked the same questions in reference to "some people".

© 2021 Daugherty SL et al. *JAMA Network Open.*

eMethods 2. Clinician-Level Clustering

The clinician each patient was scheduled to see at their enrollment visit was collected as free text by the study staff. Clinician names were cleaned and standardized prior to analysis. When clinician name was not recorded (125 of 960 participants), the clinic at which the patient was seen (out of 11 clinics) was used instead. For the diastolic blood pressure secondary outcome, full models with random effects for clinician in addition to patient-level random effects and fixed effects for site did not converge, and the clinician random effect was removed.

© 2021 Daugherty SL et al. *JAMA Network Open.*

eTable. Characteristics of the Eligible, Not Enrolled to the Enrolled Study Population

	**Eligible, not enrolled n=19,817**	**Enrolled n=960**	**p-value**
Age at eligibility or enrollment (Mean, SD)	63.00 (53.00-72.00)	64.00 (56.00-72.00)	0.07
Female sex	61.2% (12,125)	60.6% (581)	0.74
Race category			<0.01
White	27.3% (5,403)	43.5% (418)	
Black/ African American	71.1% (14,080)	54.3% (521)	
Other/ Unknown/ Multiracial	1.7% (334)	2.2% (21)	
Census tract: Median household income (X $1,000); (Median, IQR)	72.02 (52.35-96.94)	51.33 (39.13-69.64)	<0.01
Census tract: Percent with college degree (Median, IQR)	36.00 (22.00-50.00)	35.00 (19.00-53.00)	0.54
Diastolic Blood Pressure (Mean mm Hg, SD)	77.00 (68.00-86.00)	78.00 (70.00-86.00)	0.02
Systolic Blood Pressure (Mean mm Hg, SD)	139.00 (130.00-152.00)	136.00 (126.00-149.00)	<0.01
Body Mass Index (Mean, SD)	30.95 (26.79-36.39)	30.79 (26.58-36.47)	0.69
Number of comorbidities (Median, IQR)	3.00 (2.00-5.00)	4.00 (2.00-6.00)	<0.01
Ischemic Heart Disease	10.9% (2,086)	15.3% (146)	<0.01
Cerebrovascular Disease	7.5% (1,427)	9.3% (89)	0.04
Cardiac Arrhythmia	14.0% (2,672)	18.1% (173)	<0.01
Heart Failure	8.1% (1,547)	11.7% (112)	<0.01
Peripheral Vascular Disease	18.7% (3,573)	20.4% (195)	0.20
Renal Failure	17.1% (3,262)	20.7% (198)	<0.01
Depression	13.7% (2,606)	23.6% (226)	<0.01
Diabetes Mellitus	31.4% (5,987)	35.9% (344)	<0.01
Current Smoker	9.8% (1,767)	13.8% (131)	<0.01
SD = Standard deviation; IQR = Interquartile range			

© 2021 Daugherty SL et al. *JAMA Network Open.*
