# Supplementary material for: Effect of Values Affirmation on Reducing Racial Differences in Adherence to Hypertension Medication: The HYVALUE Randomized Clinical Trial
Source: JAMA Netw Open. 2021 Dec 16;4(12):e2139533. doi: 10.1001/jamanetworkopen.2021.39533 (PMC8678693; doi:10.1001/jamanetworkopen.2021.39533)
Supplement: Supplement 3. — Data Sharing Statement [file jamanetwopen-e2139533-s003.pdf]

Data Sharing Statement

Daugherty. Effect of Values Affirmation on Reducing Racial Differences in Adherence to Hypertension Medication. *JAMA Netw Open*. Published December 16, 2021. doi:10.1001/jamanetworkopen.2021.39533

Data
Data available: No

Additional Information
Explanation for why data not available: We are able to share deindentified patient-level data prospectively collected for the purposes of the trial. This data includes trial collected blood pressures, patient surveys and demographic information. In accordance with our data use agreement with participating health systems, data provided to us from their respective electronic health records (i.e. pharmacy fill data, co-morbidities) require a data use agreement between the user and the respective health system.
